# Development and Validation of a Machine Learning Score for Readmissions After Transcatheter Aortic Valve Implantation

**DOI:** 10.1016/j.jacadv.2022.100060

**Published:** 2022-08-26

**Authors:** Samian Sulaiman, Akram Kawsara, Abdulah Amr Mahayni, Abdullah El Sabbagh, Mandeep Singh, Juan Crestanello, Rajiv Gulati, Mohamad Alkhouli

**Affiliations:** a Department of Cardiovascular Disease, Mayo Clinic, Rochester, Minnesota, USA; b Division of Cardiology, West Virginia University, Morgantown, West Virginia, USA; c Department of Cardiovascular Disease, Mayo Clinic, Jacksonville, Florida, USA; d Department of Cardiac Surgery, Mayo Clinic, Rochester, Minnesota, USA

**Keywords:** machine learning, National Readmission Database, readmission, transcatheter aortic valve implantation

## Abstract

**Background:**

Identifying predictors of readmissions after transcatheter aortic valve implantation (TAVI) is an important unmet need.

**Objectives:**

We sought to explore the role of machine learning (ML) in predicting readmissions after TAVI.

**Methods:**

We included patients who underwent TAVI between 2016 and 2019 in the Nationwide Readmission Database. A total of 917 candidate predictors representing all International Classification of Diseases, Tenth Revision, diagnosis and procedure codes were included. First, we used lasso regression to remove noninformative variables and rank informative ones. Next, we used an unsupervised ML model (K-means) to identify patterns/clusters in the data. Furthermore, we used Light Gradient Boosting Machine and Shapley Additive exPlanations to specify the impact of individual predictors. Finally, we built a parsimonious model to predict 30-day readmission.

**Results:**

A total of 117,398 and 93,800 index TAVI hospitalizations were included in the 30- and 90-day analyses, respectively. Lasso regression identified 138 and 199 informative predictors for the 30- and 90-day readmission, respectively. Next, K-means recognized 2 distinct clusters: low risk and high risk. In the 30-day cohort, the readmission rate was 10.1% in the low risk group and 23.3% in the high risk group. In the 90-day cohort, the rates were 17.4% and 35.3%, respectively. The top predictors were the length of stay, frailty score, total discharge diagnoses, acute kidney injury, and Elixhauser score. These predictors were incorporated into a risk score (TAVI readmission score), which exhibited good performance in an external validation cohort (area under the curve 0.74 [0.7-0.78]).

**Conclusions:**

ML methods can leverage widely available administrative databases to identify patients at risk for readmission after TAVI, which could inform and improve post-TAVI care.

Transcatheter aortic valve implantation (TAVI) has become the dominant treatment modality for severe aortic stenosis in the United States.^1^ Despite the temporal improvement in outcomes of TAVI, readmission rates remain high.^2^ Identifying risk factors for post-TAVI readmissions could prompt additional mitigation strategies, which may improve outcomes. Previous studies that explored the issue of rehospitalizations in the TAVI population were heterogeneous and noncontemporaneous and yielded mixed results.^3^, ^4^, ^5^, ^6^, ^7^, ^8^ In addition, these studies often used institutional databases, which limit their generalizability. Machine learning (ML) has recently gained growing recognition as a potential method of improving risk stratification using the increasingly used administrative databases in cardiovascular medicine.^9^, ^10^, ^11^, ^12^, ^13^ ML algorithms have been shown to provide excellent risk discrimination regarding in-hospital mortality after TAVI.^9^ However, to our knowledge, their utility in identifying risk factors for readmission has not been previously investigated. To address this knowledge gap, we sought to develop and validate an ML-based model to predict the risk of readmission after TAVI using a nationwide contemporary administrative database.

## Methods

### Data source

We used the National Readmission Database (NRD) from January 1, 2016, through December 31, 2019, for the development stage (training data set), and the 2020 Maryland State Inpatient Database (SID) for the validation stage (testing data set).

The NRD and SID are developed by the Healthcare Cost and Utilization Project. The SIDs encompass ∼97% of all hospitalizations in the participating states, and the NRD represents a ∼60% sample of the SIDs across 30 states. The NRD and SID provide demographics, inpatient diagnoses and procedures, total costs, primary payers, length of stay, and hospital characteristics. In addition, they contain a patient linkage number that identifies discharges belonging to the same individual within the same state.^14^ Previous studies have used NRD extensively to study trends and outcomes of aortic valve interventions in the United States.^1^^,^^2^^,^^15^, ^16^, ^17^, ^18^ Because NRD and SID are publicly available and deidentified, this study was deemed exempt by the institutional review board. The data could not be made directly available for replication of the study’s results by the authors but can be obtained directly from the Healthcare Cost and Utilization Project with the appropriate data user agreement.

### Study population

**Figure 1:**
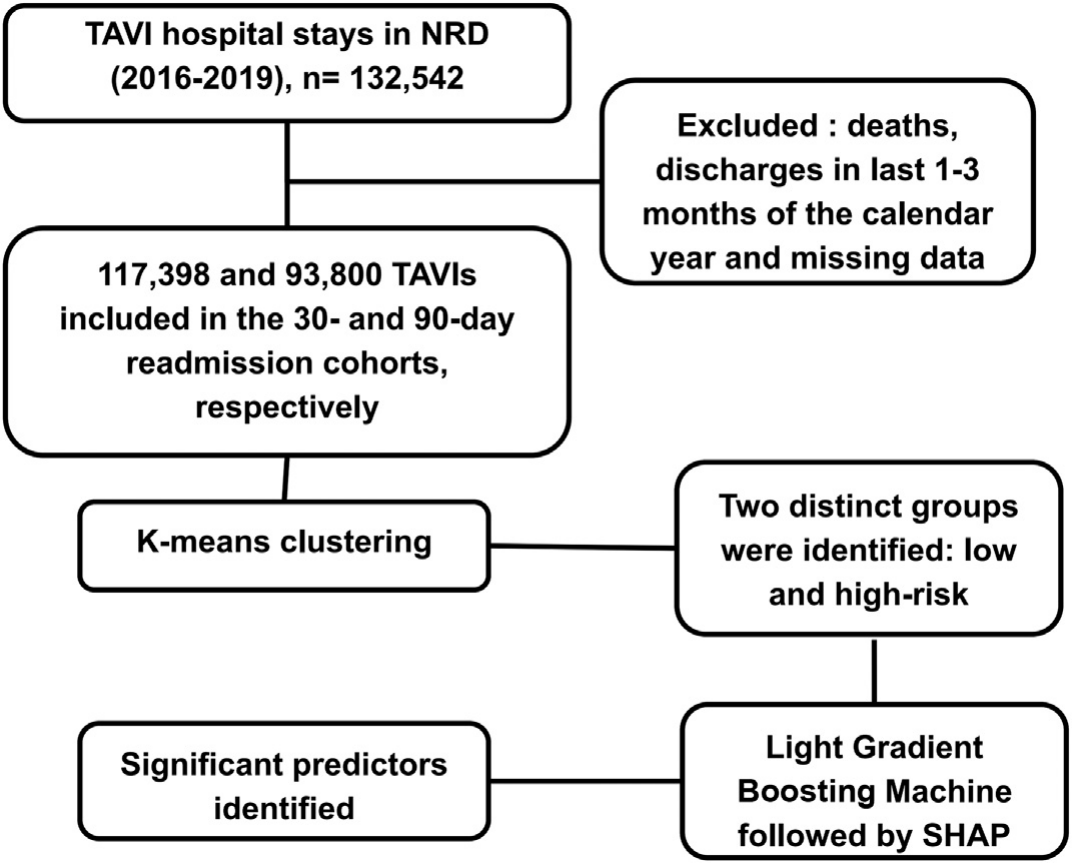
**StudyFlowchart**

We identified hospital stays for adult patients (aged ≥ 18 years) who underwent TAVI using the International Classification of Diseases-10th Revision-Clinical Modification, codes (Supplemental Table 1). We excluded patients who died, those discharged in December for the 30-day cohort and in October, November, or December for the 90-day cohort, and those who had missing information (Figure 1).

### Primary outcomes

The outcomes of interest included 30- and 90-day readmission after TAVI.

### Predictors selection process

Lasso regression was used to find the most informative predictors and rank them in descending order of importance. Nine hundred seventeen variables spanning all body systems were included in lasso regression: 49 representing demographics, Elixhauser comorbidities, and hospital stay characteristics, 542 Healthcare Cost and Utilization Project Clinical Classification Software (CCS) diagnoses, and 326 CCS procedures (Supplemental Table 2).^19^ CCS diagnoses and procedure codes aggregate all International Classification of Diseases, 10th Revision, codes into clinically meaningful categories.

### Unsupervised ML (K-means)

K-means identifies hospital stays of similar features and assigns them to clusters. The machine had the freedom to split data into 2 to 8 distinct clusters. The final number of clusters was chosen based on the largest Silhouette scores (Supplemental Figure 1). Silhouette scores represent the distance between clusters, and the larger the score, the more distant the clusters are. To visualize these clusters, we ran a principal component analysis. Then we scatter plotted hospital stays on the first and second principal components.

### Evaluating individual predictor impact

To understand which predictors K-means used to cluster hospital stays, we used a Light Gradient Boosted Machine followed by Shapley Additive exPlanations (SHAP). The resultant SHAP values represent each predictor’s impact in distinguishing K-means clusters. Orange Data Mining software^20^ and Python 3.8.9 programming language^21^ (sci-kit-learn library^22^) were used for ML models.

### Parsimonious model for clinical use

To build a model that is easy to use in clinical practice, we used the 5 top predictors with their SHAP values as coefficients. For external validation, we tested the model on the 2020 Maryland SID.

### Statistical analysis

Categorical variables were presented as percentages. Continuous variables with a symmetric distribution (eg, age) were presented as mean ± standard deviation, whereas those with a nonnormal distribution were presented as median (interquartile range). Standardized differences (SDs) of means, medians, and proportions were calculated for continuous and categorical variables. We did not compute missing data because it represented <2% of the entire data. STATA 16 was used for statistical analysis.^23^

## Results

### Baseline characteristics of the study cohorts

A total of 132,542 TAVI hospital stays between 2016 and 2019 were initially retrieved. After applying exclusion criteria, the 30-day cohort included 117,398, and the 90-day cohort included 93,800 TAVIs. The 30-day and 90-day readmission rates were 12.4% and 21.5%, respectively. In the 30-day cohort, the no readmission and readmitted groups were the same age (79.5 vs 80 years, SD = 0.06) and had similar gender distribution (female 45.2% vs 45.5%, SD = 0.01). The SDs between the 2 groups were mostly small (SD < 0.2) in all cardiovascular and noncardiovascular comorbidities except for renal failure (19.4% vs 28.4%, SD = 0.22) and iron deficiency anemia (30.9% vs 43.0%, SD = 0.26; Table 1). Very similar findings were observed in comparing the readmission vs no readmission groups in the 90-day cohort (Table 2).

**Table 1:** Comparison of Baseline Characteristics Stratified by 30-Day Readmission Status and K-Means Recognized Clusters

	No Readmission, n = 102,863 (87.6%)	Readmission, n = 14,535 (12.4%)	SD	Cluster 1, n = 97,341 (Readmission Rate 10.1%)	Cluster 2, n = 20,057 (Readmission Rate 23.3%)	SD
Demographics and hospital characteristics						
Age, y	79.5 ± 8.4	80.0 ± 8.5	0.06	79.2 ± 9.3	79.6 ± 8.2	0.05
Female	45.2%	45.5%	0.01	44.9%	47.1%	0.04
Lowest quartile household income	19.3%	19.7%	0.01	19.3%	19.9%	0.01
Medicare/Medicaid insurance	91.3%	92.9%	0.06	91.3%	92.1%	0.03
Hospital TAVR volume	132.0 (79.0-236.0)	127.0 (76.0-215.0)	0.02	139.0 (80.0-245.0)	128.0 (78.0-224.0)	0.02
Large bed size	74.6%	74.6%	0.00	74.2%	76.7%	0.06
Teaching status	88.6%	88.4%	−0.01	88.3%	89.6%	0.04
Urban location	93.5%	94.0%	0.02	93.3%	94.7%	0.06
Clinical risk factors						
Cardiovascular comorbidities						
Arrhythmia^a^	50.1%	59.7%	0.19	48.2%	66.1%	0.38
Congestive heart failure^a^	0.8%	1.6%	0.08	0.1%	4.7%	0.24
CVA (ischemic or hemorrhagic)	1.6%	2.3%	0.06	0.6%	6.8%	0.27
Diabetes without chronic complications^a^	16.1%	13.5%	−0.07	17.2%	9.1%	−0.27
Diabetes with chronic complications^a^	20.8%	28.0%	0.17	18.7%	36.4%	0.38
Hypertension^a^	8.9%	6.7%	−0.08	9.2%	5.6%	−0.15
Myocardial infarction	2.5%	4.0%	0.09	0.7%	12.6%	0.39
Obesity^a^	19.6%	19.3%	−0.01	19.4%	20.3%	0.02
Peripheral vascular disease^a^	21.6%	25.3%	0.09	20.3%	30.9%	0.23
Pulmonary circulation disorders^a^	0.0%	0.0%	0.00	0.0%	0.1%	0.04
Valvular disease^a^	0.9%	1.7%	0.09	0.1%	5.1%	0.25
Noncardiovascular comorbidities						
Alcohol abuse^a^	1.2%	1.2%	0.00	1.0%	2.2%	0.09
Blood loss anemia^a^	1.0%	1.4%	0.04	0.7%	2.5%	0.12
Chronic pulmonary disease^a^	27.1%	33.0%	0.13	25.7%	37.9%	0.26
Coagulation deficiency^a^	11.3%	14.0%	0.08	8.8%	25.4%	0.40
Depression^a^	7.8%	9.1%	0.05	7.2%	11.7%	0.15
Deficiency anemias^a^	19.4%	28.4%	0.22	15.9%	42.5%	0.56
Hyponatremia	3.7%	7.6%	0.19	1.0%	19.5%	0.51
Hypothyroidism^a^	19.6%	20.4%	0.02	19.2%	22.0%	0.07
Lymphoma^a^	1.0%	1.3%	0.02	1.0%	1.6%	0.05
Metastatic cancer^a^	0.7%	1.0%	0.04	0.6%	1.3%	0.07
Liver disease^a^	3.0%	3.7%	0.04	2.6%	5.7%	0.14
Paralysis^a^	2.4%	3.2%	0.05	1.5%	7.0%	0.23
Pneumonia	60.1%	57.7%	−0.05	60.7%	55.3%	−0.11
Renal failure^a^	30.9%	43.0%	0.26	27.3%	57.5%	0.62
Solid tumor without metastasis^a^	2.4%	3.1%	0.05	2.3%	3.5%	0.07
Septicemia	0.6%	1.5%	0.10	0.0%	4.1%	0.23
Weight loss^a^	2.5%	4.8%	0.13	1.3%	10.2%	0.32

Values are mean ± standard deviation, %, or median (IQR).

SD = standardized difference.

a Defined by HCUP Elixhauser comorbidities software.

**Table 2:** Comparison of Baseline Characteristics Stratified by 90-Day Readmission Status and K-Means Recognized Clusters

	No Readmission, n = 73,652 (78.5%)	Readmission, n = 20,148 (21.5%)	SD	Cluster 1, n = 72,441 (Readmission Rate 17.4%)	Cluster 2, n = 21,359 (Readmission Rate 35.3%)	SD
Demographics and hospital characteristics						
Age, y	79.5 ± 8.4	79.9 ± 8.5	0.05	79.7 ± 8.2	79.5 ± 9.0	−0.02
Female	45.0%	46.0%	0.02	45.4%	44.8%	−0.01
Lowest quartile household income	19.3%	19.8%	0.01	19.2%	20.0%	0.02
Medicare/Medicaid insurance	91.3%	92.8%	0.05	91.4%	92.6%	0.04
Hospital TAVR volume median (IQR)	134.0 (80.0-237.0)	127.0 (78.0-220.0)	0.02	129.0 (79.0-233.0)	139.0 (81.0-241.0)	0.02
Large bed size	74.7%	74.5%	−0.01	74.3%	75.9%	0.04
Teaching status	88.7%	88.1%	−0.02	88.3%	89.6%	0.04
Urban location	93.5%	94.1%	0.03	93.3%	94.8%	0.06
Clinical risk factors						
Cardiovascular comorbidities						
Arrhythmia^a^	49.5%	59.6%	0.20	47.1%	67.0%	0.40
Congestive heart failure^a^	0.7%	1.5%	0.08	0.1%	3.5%	0.37
CVA (ischemic or hemorrhagic)	1.6%	2.2%	0.05	0.6%	5.3%	0.36
Diabetes without chronic complications^a^	16.5%	14.4%	−0.06	18.1%	9.1%	−0.25
Diabetes with chronic complications^a^	19.9%	27.2%	0.18	16.6%	37.9%	0.53
Hypertension^a^	8.6%	6.6%	−0.07	9.1%	5.1%	−0.15
Myocardial infarction	2.4%	3.8%	0.08	0.8%	9.0%	0.52
Obesity^a^	19.6%	19.4%	0.00	19.4%	20.0%	0.02
Peripheral vascular disease^a^	21.6%	24.7%	0.07	19.9%	30.4%	0.25
Pulmonary circulation disorders^a^	0.0%	0.0%	−0.01	0.0%	0.1%	0.07
Valvular disease^a^	0.8%	1.6%	0.08	0.1%	3.8%	0.38
Noncardiovascular comorbidities						
Alcohol abuse^a^	1.2%	1.3%	0.01	1.0%	2.0%	0.10
Blood loss anemia^a^	1.0%	1.4%	0.04	0.8%	2.1%	0.12
Chronic pulmonary disease^a^	26.9%	32.8%	0.13	24.4%	41.0%	0.37
Coagulation deficiency^a^	11.2%	13.9%	0.08	8.7%	22.0%	0.42
Depression^a^	7.6%	9.0%	0.05	7.0%	11.0%	0.15
Deficiency anemias^a^	18.8%	27.6%	0.22	14.0%	43.7%	0.77
Hyponatremia	3.5%	6.7%	0.16	1.4%	13.7%	0.64
Hypothyroidism^a^	19.5%	20.4%	0.02	18.9%	22.4%	0.09
Lymphoma^a^	1.0%	1.4%	0.04	0.9%	1.8%	0.08
Metastatic cancer^a^	0.6%	1.0%	0.05	0.5%	1.3%	0.10
Liver disease^a^	2.9%	3.8%	0.05	2.3%	5.9%	0.21
Paralysis^a^	2.3%	3.1%	0.05	1.3%	6.6%	0.35
Pneumonia	59.9%	58.0%	−0.04	60.7%	55.5%	−0.10
Renal failure^a^	30.1%	41.8%	0.25	23.5%	63.8%	0.92
Solid tumor without metastasis^a^	2.1%	3.4%	0.08	2.1%	3.3%	0.08
Septicemia	0.6%	1.3%	0.08	0.0%	3.0%	0.36
Weight loss^a^	2.3%	4.5%	0.13	1.1%	8.6%	0.47

Values are mean ± standard deviation, %, or median (IQR).

SD = standardized difference.

a Defined by HCUP Elixhauser comorbidities software.

### K-means clustering

**Figure 2:**
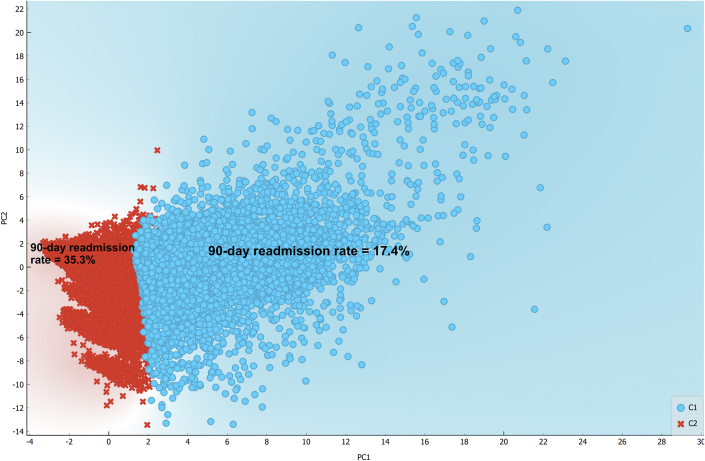
**Scatterplot of the 90-Day Cohort on Principal Components 1 and 2** C1 = low-risk cluster; C2 = high-risk cluster.

In the 30-day cohort, Lasso regression ranked 138 informative predictors in descending order of importance (Supplemental Table 3). Next, K-means found 2 clusters; a large cluster that included 97,341 TAVIs with a readmission rate of 10.1% (95 CI: 9.9%-10.3%) and a smaller cluster that included 20,057 TAVIs with a readmission rate of 23.3% (22.7%-23.9%; Central Illustration). Similarly, in the 90-day cohort, Lasso regression identified 199 informative predictors (Supplemental Table 4), and K-means identified 2 clusters: a low-risk cluster (n = 72,441) with a readmission rate of 17.4% (17.1%-17.7%) and a high-risk cluster (n = 21,359) with a readmission rate of 35.3% (34.7%-35.9%) (Figure 2).

**Central Illustration:**
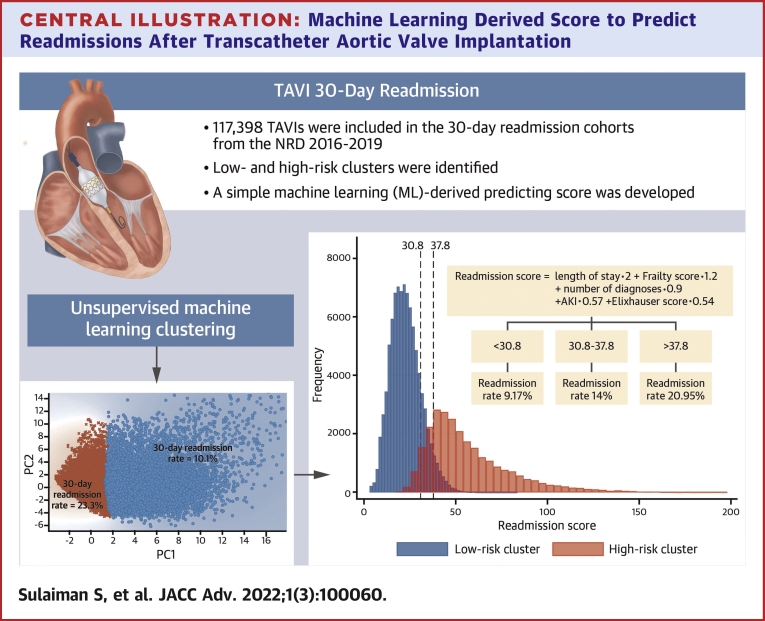
Machine Learning Derived Score to Predict Readmissions After Transcatheter Aortic Valve Implantation The **right lower subfigure** shows the scatterplot of the 30-day cohort on principal components 1 and 2; **blue dots** represent the low-risk cluster, and **red crosses** represent the high-risk cluster. The **left lower subfigure** shows the suggested score equation with a histogram of the score per high and low-risk clusters; 37.8 is the 95th percentile of the low-risk cluster, and 30.8 is the fifth percentile of the high-risk cluster. AKI = acute kidney injury; NRD = Nationwide Readmission Database; PC = principal component; TAVI = transcatheter aortic valve implantation.

### Comparison of high- and low-risk clusters

**Figure 3:**
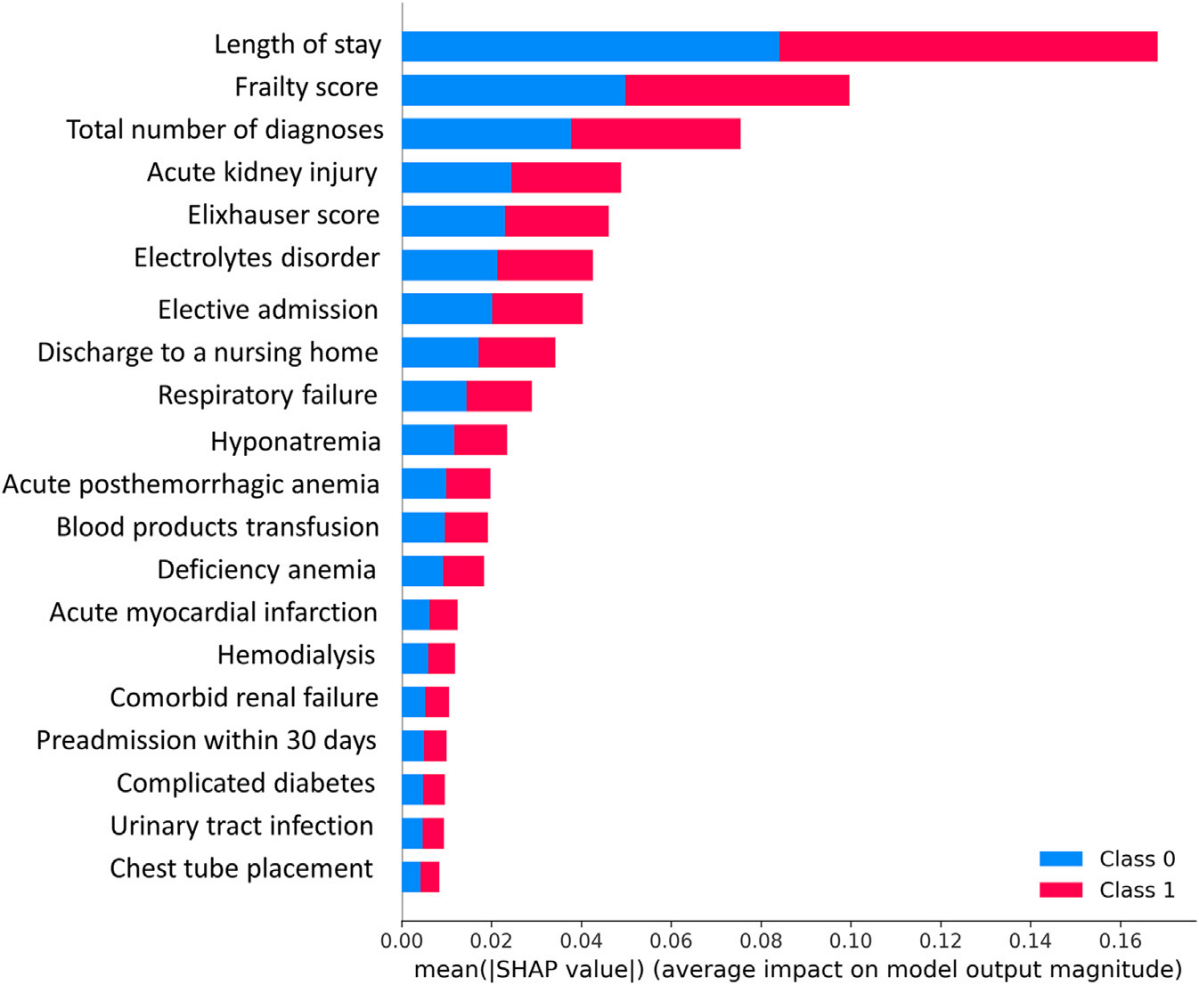
**SHAP Values of the Top 20 Predictors of Light GBM Model** Class 0 = low risk group; Class 1 = high risk group.

**Figure 4:**
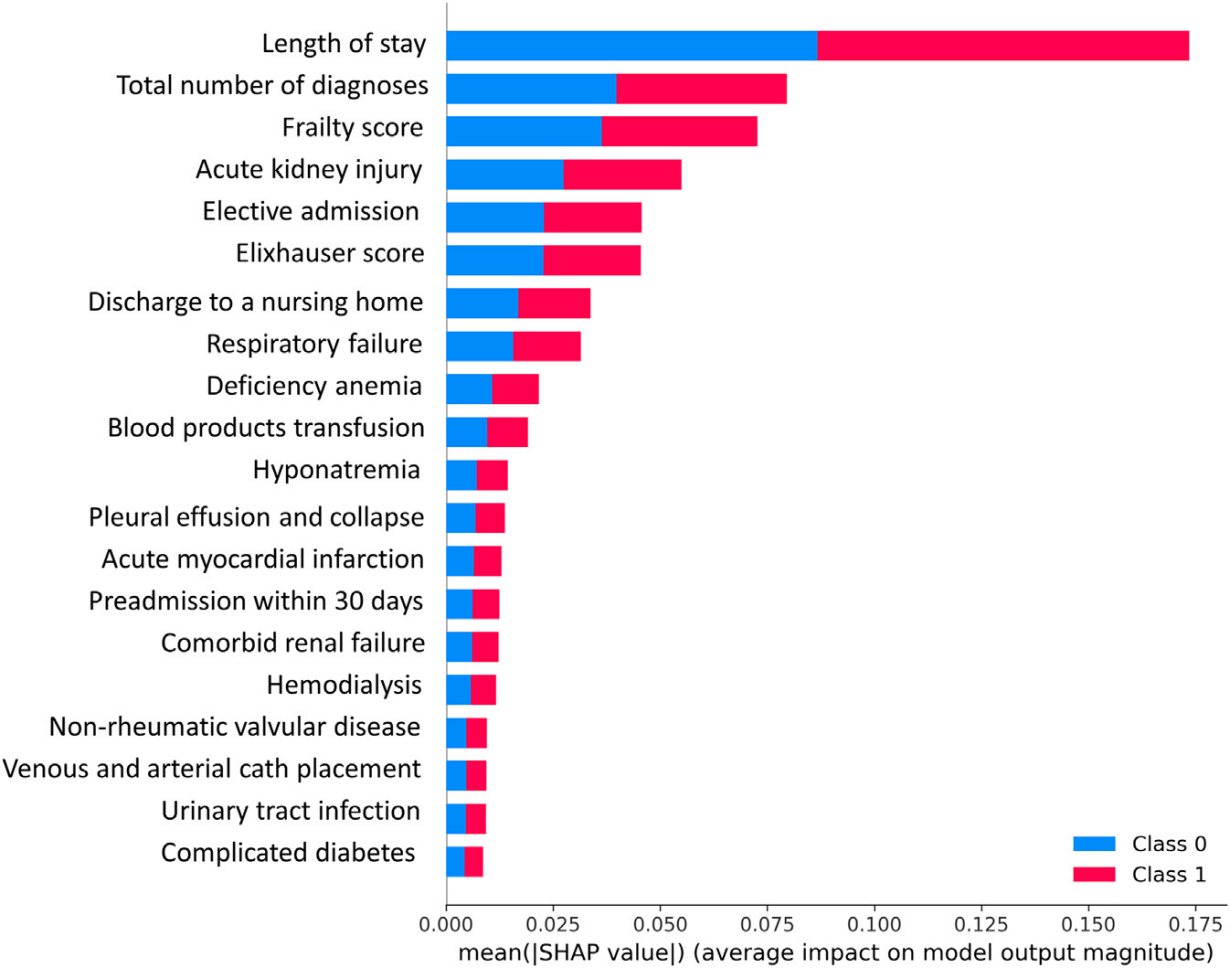
**SHAP Values of the Top 20 Predictors of Light GBM Model** Class 0 = low risk group; Class 1 = high risk group.

A comparison between the baseline characteristics of the high risk and low risk groups is shown in Tables 1 and 2. The top 20 predictors with the highest SHAP values are displayed in Figures 3 and 4. The frequencies of these predictors in the high risk and low risk groups are shown in Tables 3 and 4. The high-risk cluster was had a higher comorbidities burden (Elixhauser score 8 [5-12] vs 2 [0-5], SD = 0.44), a higher total number of diagnoses (23 [18-27] vs 15 [11-18], SD = 0.55), and a higher prevalence of hemodialysis (11.1% vs 0.8%, SD = 0.61). Although both groups had a similar age (79.6 [8.2] vs 79.2 [9.3], SD = 0.05), the high risk group had a higher frailty score (7.2 [4.9-10.9] vs 2.1 [1.4-3.7] vs SD = 0.56). The high risk group also had a more extended hospital stay (8 [4-14] days vs 2 [1-3] days, SD = 0.57) and higher rates of complications, including anemia requiring transfusion (16.8% vs 2.6%, SD = 0.62), acute kidney injury (AKI) (34.3% vs 2.6%, SD = 1.19), respiratory failure (23.2% vs 2.6%, SD = 0.84), hyponatremia (13.7% vs 1.4% SD = 0.64), and acute myocardial infarction (9.0 vs 0.8%, SD = 0.52). Discharges to a nursing facility were more frequent in the high risk group (36.3% vs 4.1%, SD = 1.12). Similar findings were observed in the 90-day readmission cohort analyses.

**Table 3:** Comparisons of the Top 20 Most Impactful Predictors of High vs Low Risk of 30-Day Readmission After TAVI

	No Readmission, n = 102,863 (87.6%)	Readmission, n = 14,535 (12.4%)	SD	Cluster 1, n = 97,341 (Readmission Rate 10.1%)	Cluster 2, n = 20,057 (Readmission Rate 23.3%)	SD
Length of stay	2.0 (1.0-4.0)	3.0 (2.0-8.0)	0.14	2.0 (1.0-3.0)	10.0 (6.0-16.0)	0.57
Frailty score	2.9 (1.4-5.1)	3.8 (1.8-7.1)	0.11	2.3 (1.4-3.9)	7.9 (5.5-11.6)	0.52
Number of diagnoses	16.0 (12.0-19.0)	18.0 (14.0-23.0)	0.12	15.0 (12.0-18.0)	25.0 (19.0-28.0)	0.50
Acute kidney injury	8.7%	17.3%	0.29	2.9%	43.0%	1.57
Elixhauser score^a^	3.0 (0.0-6.0)	5.0 (0.0-8.0)	0.10	2.0 (0.0-5.0)	8.0 (5.0-13.0)	0.39
Electrolytes disorders	13.5%	22.6%	0.26	6.5%	54.0%	1.55
Elective admission	82.6%	73.2%	−0.24	89.5%	42.7%	−1.35
Discharge to a skilled nursing facility	10.0%	19.3%	0.30	5.5%	38.7%	1.15
Respiratory failure	6.4%	12.7%	0.24	2.4%	30.3%	1.18
Hyponatremia	3.7%	7.6%	0.20	1.0%	19.5%	0.98
Hemorrhagic anemia	11.7%	16.6%	0.15	8.5%	30.5%	0.69
Blood and other products transfusion	5.2%	10.3%	0.22	2.8%	20.2%	0.77
Deficiency anemia	19.4%	28.4%	0.23	15.9%	42.5%	0.68
Myocardial infarction	2.5%	4.0%	0.09	0.7%	12.6%	0.76
Hemodialysis	2.6%	6.4%	0.22	1.5%	11.2%	0.57
Renal failure^a^	30.9%	43.0%	0.26	27.3%	57.5%	0.66
Preadmitted in 30 d	11.4%	19.7%	0.25	10.2%	22.9%	0.39
DM with complications	20.8%	28.1%	0.18	18.7%	36.4%	0.43
Urinary tract infection	4.8%	7.8%	0.14	2.7%	17.3%	0.68
Chest tube placement	1.7%	3.8%	0.15	0.5%	9.2%	0.64

Values are median (IQR) or %.

a Defined by HCUP Elixhauser comorbidities software.

**Table 4:** Comparisons of the Top 20 Most Impactful Predictors of High vs Low Risk of 90-Day Readmission After TAVI

	No Readmission, n = 73,652 (78.5%)	Readmission, n = 20,148 (21.5%)	SD	Cluster 1, n = 72,441 (Readmission Rate 17.4%)	Cluster 2, n = 21,359 (Readmission Rate 35.3%)	SD
Length of stay	2 (1-4)	3 (2-7)	0.16	2 (1-3)	8 (4-14)	0.57
Number of diagnoses	16 (12-19)	18 (14-23)	0.14	15 (11-18)	23 (18-27)	0.55
Frailty score	2.8 (1.4-5)	3.7 (1.6-6.8)	0.13	2.1 (1.4-3.7)	7.2 (4.9-10.9)	0.56
Acute kidney injury	8.2%	15.7%	0.25	2.6%	34.3%	1.19
Elective admission	83.1%	74.6%	−0.22	91%	48.4%	−1.23
Elixhauser score^a^	3 (0-6)	5 (0-8)	0.12	2 (0-5)	8 (5-12)	0.44
Discharge to a skilled nursing facility	9.4%	18.8%	0.30	4.1%	36.3%	1.12
Respiratory failure	6%	11.8%	0.22	2.6%	23.2%	0.84
Deficiency anemia	18.8%	27.6%	0.22	14%	43.7%	0.77
Blood transfusion	4.9%	9.4%	0.19	2.6%	16.8%	0.62
Hyponatremia	3.5%	6.7%	0.16	1.4%	13.7%	0.64
Pleural effusion and collapse	4.9%	7.7%	0.12	3.2%	13.3%	0.45
Myocardial Infarction	2.4%	3.8%	0.08	0.8%	9%	0.52
Preadmitted in 30 d	10.9%	18.6%	0.24	9.3%	23.6%	0.44
Renal failure^a^	30.1%	41.8%	0.25	23.5%	63.8%	0.92
Hemodialysis	2.4%	5.8%	0.20	0.8%	11.1%	0.61
Nonrheumatic valve disease	89%	87.3%	−05	89.7%	84.8%	−0.16
Venous and arterial catheter placement	6.9%	9.5%	0.10	4.9%	15.9%	0.42
Urinary tract infection	4.5%	7.6%	0.14	2.6%	13.8%	0.52
Diabetes with complications^a^	19.9%	27.2%	0.18	16.6%	37.9%	0.53

Values are median (IQR) or %.

IQR = interquartile range; SD = standardized difference.

a Defined by HCUP Elixhauser comorbidities software.

### Parsimonious model for predicting 30-day readmission after TAVI

Based on SHAP values (Figure 3), we built a simple risk score; The readmission score = length of stay × 2 + frailty score × 1.2 + number of diagnoses × 0.9 + AKI × 0.57 + Elixhauser score × 0.54. The histograms of the readmission score stratified by the high- and low-risk clusters are shown in the Central Illustration. The 95th percentile of the low-risk cluster (37.8) and the fifth percentile cutoff of the high-risk cluster were used to classify patients into high risk, intermediate risk, and low risk groups (Supplemental Figure 2): readmission score >37.8 (high risk) corresponded with a readmission rate of 20.95%; readmission score <30.8 (low risk) corresponded with a readmission rate of 9.17%; readmission score 30.8 to 37.8 (intermediate risk) corresponded with a readmission rate of 14%.

**Figure 5:**
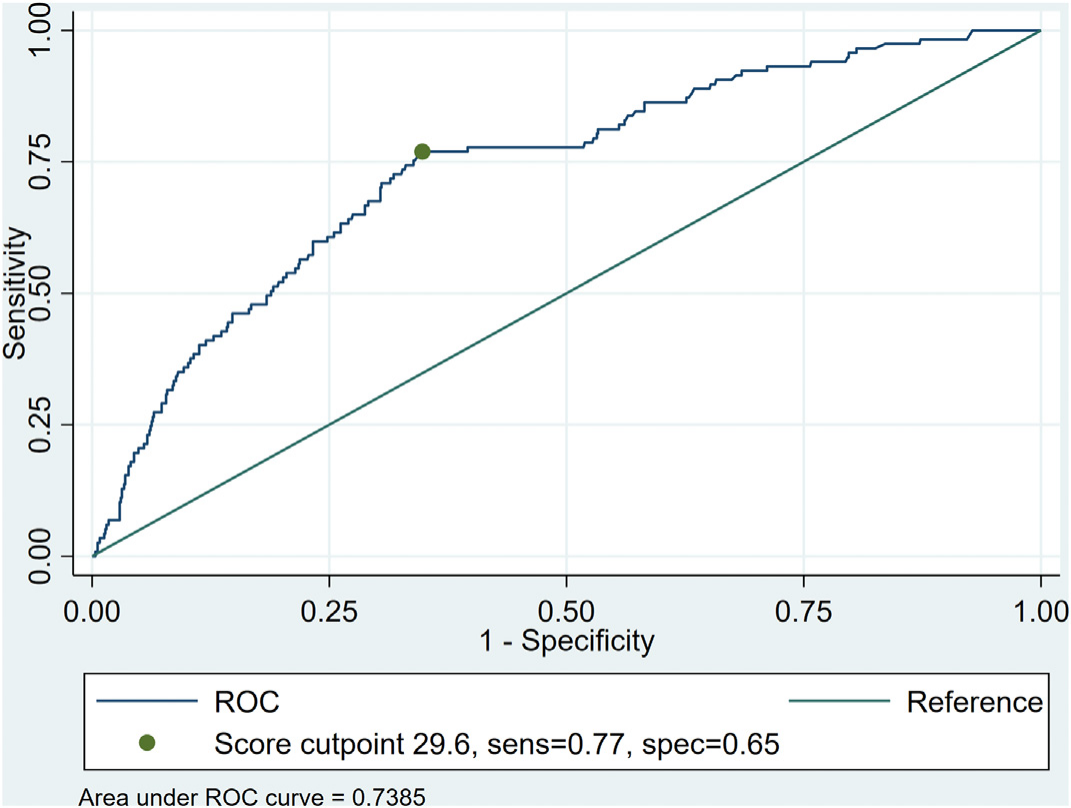
**Receiver Operator Curve and Optimal Cutpoint for the TAVI 30-Day Readmission Score in the ValidationCohort**

Next, we applied these cutoffs to Maryland SID 2020. Maryland SID contained 976 index TAVI hospitalizations, with patients surviving to discharge. The validation data set was comparable to the development data set in key baseline characteristics, demographics, predictors, and rate of readmission (Supplemental Table 5). In this external validation cohort, the model had a good performance in identifying patients who were readmitted with an area under the curve = 0.74 (0.7-0.78) (Figure 5). When applying the TAVI readmission score to predict high vs intermediate vs low-risk “clusters,” the model correctly identified all patients in the corresponding categories:

(A) high-risk cluster: 251 TAVI patients with a score >37.82 and a readmission rate of 25.5% (within a 95% CI of the predicted rate of 20.95% [15.9%-26%])
(B) low-risk cluster: 616 TAVI patients had a score <30.78, and a readmission rate of 5.19% (below the 95% CI of the predicted rate of 9.17% [6.9%-11.4%])
(C) intermediate-risk cluster: 109 TAVI patients had a score between 30.7 and 37.8 and a readmission rate of 19.27% (within the 95% CI of the predicted rate of 14% [7.5%-20.5%]).

## Discussion

This study proposes a simple ML-derived score to predict unplanned rehospitalizations after TAVI using widely available administrative data (Central Illustration). The “readmission score” could be incorporated into the electronic medical records to automatically flag patients at high risk for readmissions after TAVI allowing targeted interventions to mitigate that risk.

Several studies investigated predictors of readmission after TAVI. Using the STS/ACC/TVT Registry database of self-expanding TAVIs between 2015 and 2017, Sanchez et al^3^ found that diabetes, atrial fibrillation, advanced heart failure symptoms, home oxygen, decreased 5-m gait speed, or inability to walk, creatinine >1.6 mg/dL, length of stay >5 days, major vascular complication, and ≥moderate postprocedure aortic or mitral valve regurgitation were predictors of 30-day readmission. Based on these predictors, patients were stratified into low risk, moderate risk, and high risk for 30-day readmission, with a large (2.5-fold) difference in readmission rates between the low risk (5.8%) and high risk groups (14.6%). Another multicenter study by Nombela-Franco et al^4^ showed that periprocedural major bleeding, anemia, reduced left ventricular ejection fraction, and concomitant antiplatelet and anticoagulation use predicted early readmission after TAVI. A third study involved 1,749 TAVIs from a Japanese multicenter registry identified atrial fibrillation, obstructive pulmonary disease, Frailty Scale ≥4, chronic kidney disease, and moderate-to-severe mitral regurgitation as independent predictors of readmission for heart failure after TAVI.^5^

The previously mentioned studies had 2 major limitations: first, they used variables that might not be routinely collected in every center (eg, 5-m gait speed), hence limiting their broad applicability; and second; none of the studies constructed or validated an actual risk score that can be easily incorporated in clinical practice. Our study sought to mitigate these limitations by: 1) using variables from administrative data sets that are routinely used in virtually all hospitals; and 2) by constructing a validating a risk score that can be incorporated within hospital databases to stratify TAVI patients with regard to their risk of readmissions. In addition, the previous studies identified dissimilar predictors of readmission, reflecting the preselection of a narrow pool of candidate predictors. Therefore, we applied ML methods that, contrary to conventional statistics, allow a full survey of all existing variables in the database (>900 in this study), hence allowing potential discovery of new predictors and avoiding priori knowledge and prejudice.

Various ML methods could be used for predictive analytics in cardiovascular medicine.^24^, ^25^, ^26^, ^27^, ^28^ When evaluating the potential ML approaches to this analysis, one important factor needed to be considered. The absolute differences in baseline characteristics and complications rate between the readmitted and no-readmission patients were relatively small. As a result, using supervised ML methods or conventional statistics such as logistic regression would result in multiple statistically significant but weak predictors of readmission. Therefore, we opted to use unsupervised ML techniques to discern more distinct groups, followed by supervised ML to find predictors of these 2 distinct groups. This method allowed the identification of 5 key predictors of the readmissions both at 30- and 90-day (length of stay, frailty score, total discharge diagnoses, AKI, and Elixhauser score). These predictors were incorporated into a risk score (TAVI 30-readmission score) that showed an excellent ability to classify patients into high risk, medium risk, and low risk of readmission. Compared with a risk tool developed by Khera et al^8^ using conventional statistics, our model had better external validation (area under the curve 0.74 vs 0.63) and used fewer predictors (5 vs 9). Finally, the main advantage of this score is that it could be derived from billing codes and automatically calculated in the electronic medical records at the time of discharge. This information may then alert the physician to patients who are at very high risk of readmission. Interventions to reduce readmissions (eg, closer or more frequent follow-up) in this group could then be implemented, although more data are needed to examine the type and efficacy of these interventions.

### Study Limitations

First, NRD does not include echocardiography, laboratory, or pharmacology information. However, the lack of this information should not deprecate our findings. For example, in a substudy of the Patient-Centered Care Transitions in HF trial, cluster analysis revealed 6 clinical phylogroups, which outperformed left ventricular ejection fraction in prognosticating all-cause death and rehospitalization at 6 and 12 months.^29^ Second, the NRD is an administrative database hindered by the limitations of this type of database, such as coding errors. Finally, further validation of the parsimonious model is needed.

## Conclusions

ML facilitated the construction of a simple score to predict readmissions after TAVI. The TAVI readmission score, derived from routinely used billing codes, could be incorporated into electronic medical records to automatically identify patients who are at elevated risk for readmissions. Further validation in larger cohorts is warranted.

## Funding support and author disclosures

The authors have reported that they have no relationships relevant to the contents of this paper to disclose.

PERSPECTIVES **COMPETENCY IN PRACTICE-BASED LEARNING AND IMPROVEMENT:** This study analyzed transcatheter aortic valve replacement readmissions on a national level and recognized clinically relevant patterns. Furthermore, we transformed our findings into a simple score that can be incorporated into electronic medical records and, therefore, be widely applicable by individual clinicians.

**TRANSLATIONAL OUTLOOK:** Prospective validation in a larger cohort would be the next step in corroborating the suggested score. In addition, future studies would be needed to compare different interventions to prevent readmission using this score.
