# Supplementary material for: Development and Validation of a Machine Learning Score for Readmissions After Transcatheter Aortic Valve Implantation
Source: JACC Adv. 2022 Aug 26;1(3):100060. doi: 10.1016/j.jacadv.2022.100060 (PMC11198219; doi:10.1016/j.jacadv.2022.100060)
Supplement: Supplement Figure 1 and Tables 1-6 [file mmc1.docx]

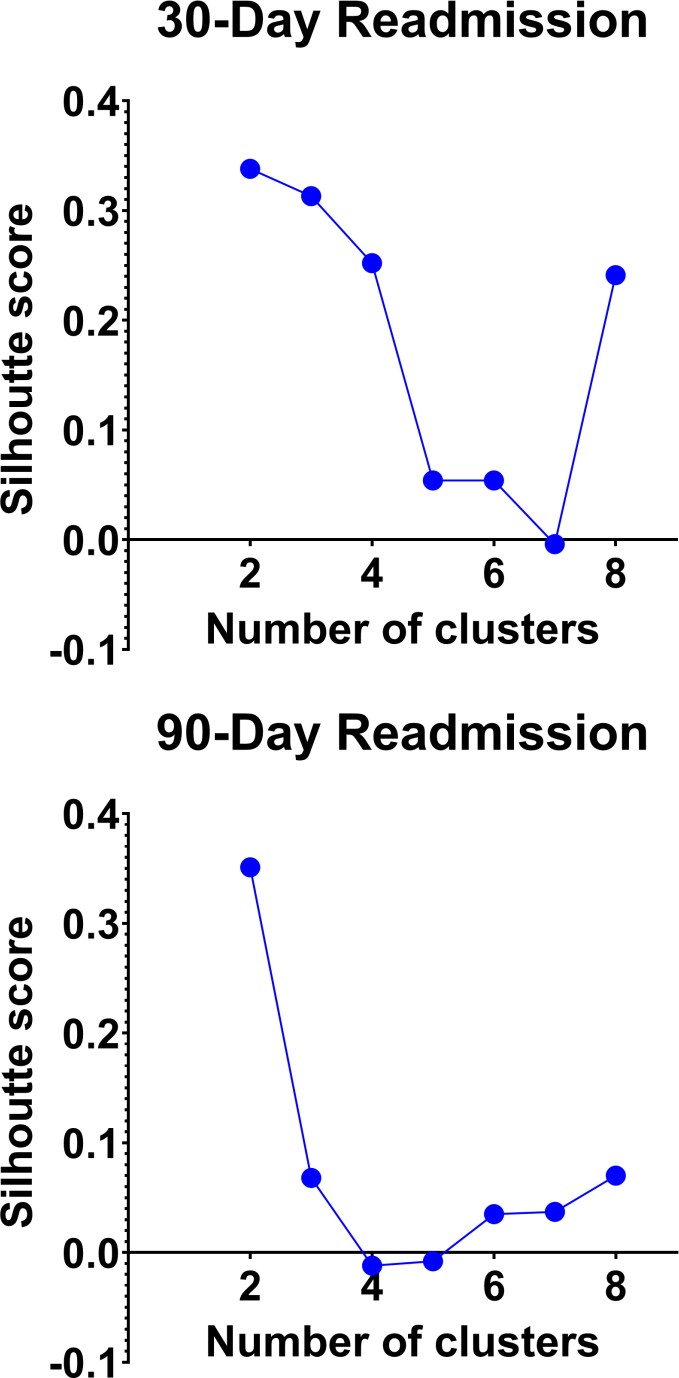


**Supplemental Figure 1:** Silhouette scores showing the number of clusters in the 30-day and 90-day readmission cohorts. Silhouette score was highest when splitting cohorts into 2 clusters

| Age |
| --- |
| Gender |
| Teaching hospital status |
| Hospital bed size |
| Hospital TAVI volume |
| Length of stay |
| Medicare/Medicaid insurance |
| Hospital location (rural vs. urban) |
| Preadmission within 30 days |
| Discharge to a skilled nursing facility |
| Leaving against medical advice |
| Hyponatremia |
| Therapeutic errors |
| Adverse drug reactions |
| Frailty score |
| Elective admission |
| **29 Elixhauser comorbidities (for details, refer to** [Tools Archive for Elixhauser Comorbidity](https://www.hcup-us.ahrq.gov/toolssoftware/comorbidityicd10/comorbidity_icd10_archive.jsp)  [Software Refined for ICD-10-CM (ahrq.gov)](https://www.hcup-us.ahrq.gov/toolssoftware/comorbidityicd10/comorbidity_icd10_archive.jsp) |
| HIV and AIDS (Acquired immune deficiency syndrome) |
| Alcohol abuse |
| Deficiency anemias |
| Rheumatoid arthritis/collagen vascular diseases |
| Blood loss anemia |
| Congestive heart failure |
| Chronic pulmonary disease |
| Coagulation deficiency |
| Depression |
| Diabetes without chronic complications |
| Diabetes with chronic complications  Drug abuse |
| Hypertension |
| Hypothyroidism |
| Liver disease |
| Lymphoma |
| Fluid and electrolyte disorders |
| Metastatic cancer |
| Other neurological disorders |
| Obesity |
| Paralysis |
| Peripheral vascular disease |
| Renal failure |
| Solid tumor without metastasis |
| Chronic peptic ulcer disease |
| Valvular disease |
| Weight loss |
| **CCSR Diagnosis Category Description (for details of ICD 10, refer to** [Clinical Classifications](https://www.hcup-us.ahrq.gov/toolssoftware/ccsr/ccs_refined.jsp)  [Software Refined (CCSR) (ahrq.gov)](https://www.hcup-us.ahrq.gov/toolssoftware/ccsr/ccs_refined.jsp) |
| Nutritional anemia |
| Hemolytic anemia |

| Aplastic anemia |
| --- |
| Acute posthemorrhagic anemia |
| Sickle cell trait/anemia |
| Coagulation and hemorrhagic disorders |
| Diseases of white blood cells |
| Immunity disorders |
| Postprocedural or postoperative complications of the spleen |
| Other specified and unspecified hematologic conditions |
| Chronic rheumatic heart disease |
| Acute rheumatic heart disease |
| Nonrheumatic and unspecified valve disorders |
| Endocarditis and endocardial disease |
| Myocarditis and cardiomyopathy |
| Pericarditis and pericardial disease |
| Essential hypertension |
| Hypertension with complications and secondary hypertension |
| Acute myocardial infarction |
| Complications of acute myocardial infarction |
| Coronary atherosclerosis and other heart disease |
| Nonspecific chest pain |
| Acute pulmonary embolism |
| Pulmonary heart disease |
| Other and ill-defined heart disease |
| Conduction disorders |
| Cardiac dysrhythmias |
| Cardiac arrest and ventricular fibrillation |
| Heart failure |
| Cerebral infarction |
| Acute hemorrhagic cerebrovascular disease |
| Sequela of hemorrhagic cerebrovascular disease |
| Occlusion or stenosis of precerebral or cerebral arteries without infarction |
| Other and ill-defined cerebrovascular disease |
| Sequela of cerebral infarction and other cerebrovascular disease |
| Peripheral and visceral vascular disease |
| Arterial dissections |
| Gangrene |
| Aortic; peripheral; and visceral artery aneurysms |
| Aortic and peripheral arterial embolism or thrombosis |
| Hypotension |
| Other specified and unspecified circulatory disease |
| Acute phlebitis; thrombophlebitis and thromboembolism |
| Chronic phlebitis; thrombophlebitis and thromboembolism |
| Varicose veins of lower extremity |
| Postthrombotic syndrome and venous insufficiency/hypertension |
| Vasculitis |
| Postprocedural or postoperative circulatory system complication |
| Other specified diseases of veins and lymphatics |
| Intestinal infection |
| Disorders of teeth and gingiva |

| Diseases of mouth; excluding dental |
| --- |
| Esophageal disorders |
| Gastroduodenal ulcer |
| Gastrointestinal and biliary perforation |
| Gastritis and duodenitis |
| Other specified and unspecified disorders of stomach and duodenum |
| Appendicitis and other appendiceal conditions |
| Abdominal hernia |
| Regional enteritis and ulcerative colitis |
| Intestinal obstruction and ileus |
| Diverticulosis and diverticulitis |
| Hemorrhoids |
| Anal and rectal conditions |
| Peritonitis and intra-abdominal abscess |
| Biliary tract disease |
| Hepatic failure |
| Other specified and unspecified liver disease |
| Pancreatic disorders (excluding diabetes) |
| Gastrointestinal hemorrhage |
| Noninfectious gastroenteritis |
| Noninfectious hepatitis |
| Postprocedural or postoperative digestive system complication |
| Other specified and unspecified gastrointestinal disorders |
| Otitis media |
| Diseases of middle ear and mastoid (except otitis media) |
| Diseases of inner ear and related conditions |
| Hearing loss |
| Postprocedural or postoperative ear and/or mastoid process complication |
| Other specified and unspecified disorders of the ear |
| Thyroid disorders |
| Diabetes mellitus without complication |
| Diabetes mellitus with complication |
| Diabetes mellitus, Type 1 |
| Diabetes mellitus, Type 2 |
| Diabetes mellitus, due to underlying condition, drug or chemical induced, or other specified type |
| Nutritional deficiencies |
| Malnutrition |
| Obesity |
| Disorders of lipid metabolism |
| Fluid and electrolyte disorders |
| Cystic fibrosis |
| Pituitary disorders |
| Postprocedural or postoperative endocrine or metabolic complication |
| Other specified and unspecified endocrine disorders |
| Other specified and unspecified nutritional and metabolic disorders |
| Sequela of malnutrition and other nutritional deficiencies |
| External cause codes: cut/pierce; initial encounter |
| External cause codes: drowning/submersion; initial encounter |
| External cause codes: fall; initial encounter |

| External cause codes: fire/burn; initial encounter |
| --- |
| External cause codes: firearm; initial encounter |
| External cause codes: machinery; initial encounter |
| External cause codes: motor vehicle traffic (MVT); initial encounter |
| External cause codes: pedal cyclist; not MVT; initial encounter |
| External cause codes: pedestrian; not MVT; initial encounter |
| External cause codes: transport; not MVT; initial encounter |
| External cause codes: natural/environment; initial encounter |
| External cause codes: bites; initial encounter |
| External cause codes: overexertion; initial encounter |
| External cause codes: poisoning by drug |
| External cause codes: poisoning by non-drug |
| External cause codes: struck by; against; initial encounter |
| External cause codes: suffocation/inhalation; initial encounter |
| External cause codes: other specified, classifiable and NEC; initial encounter |
| External cause codes: unspecified mechanism |
| External cause codes: intent of injury, accidental/unintentional |
| External cause codes: intent of injury, self-harm |
| External cause codes: intent of injury, assault |
| External cause codes: intent of injury, undetermined |
| External cause codes: intent of injury, legal intervention/war |
| External cause codes: complications of medical and surgical care, initial encounter |
| External cause codes: activity codes |
| External cause codes: place of occurrence of the external cause |
| External cause codes: evidence of alcohol involvement |
| External cause codes: subsequent encounter |
| External cause codes: sequela |
| Cornea and external disease |
| Cataract and other lens disorders |
| Glaucoma |
| Uveitis and ocular inflammation |
| Retinal and vitreous conditions |
| Neuro-ophthalmology |
| Strabismus |
| Oculofacial plastics and orbital conditions |
| Refractive error |
| Blindness and vision defects |
| Postprocedural or postoperative eye complication |
| Other specified eye disorders |
| Encounter for administrative purposes |
| Encounter for mental health services related to abuse |
| Encounter for observation and examination for conditions ruled out (excludes infectious disease, neoplasm, mental disorders) |
| Encounter for prophylactic or other procedures |
| Encounter for prophylactic measures (excludes immunization) |
| Encounter for antineoplastic therapies |
| Encounter for mental health conditions |
| Neoplasm-related encounters |
| Implant, device or graft related encounter |

| Other aftercare encounter |
| --- |
| Counseling related to sexual behavior or orientation |
| Other specified encounters and counseling |
| Contraceptive and procreative management |
| Medical examination/evaluation |
| Resistance to antimicrobial drugs |
| Exposure, encounters, screening or contact with infectious disease |
| No immunization or underimmunization |
| Screening for neurocognitive or neurodevelopmental condition |
| Socioeconomic/psychosocial factors |
| Lifestyle/life management factors |
| Personal/family history of disease |
| Acquired absence of limb or organ |
| Organ transplant status |
| Carrier status |
| Other specified status |
| Nephritis; nephrosis; renal sclerosis |
| Acute and unspecified renal failure |
| Chronic kidney disease |
| Urinary tract infections |
| Calculus of urinary tract |
| Other specified and unspecified diseases of kidney and ureters |
| Other specified and unspecified diseases of bladder and urethra |
| Urinary incontinence |
| Hematuria |
| Proteinuria |
| Vesicoureteral reflux |
| Hyperplasia of prostate |
| Inflammatory conditions of male genital organs |
| Erectile dysfunction |
| Male infertility |
| Other specified male genital disorders |
| Nonmalignant breast conditions |
| Inflammatory diseases of female pelvic organs |
| Endometriosis |
| Prolapse of female genital organs |
| Menstrual disorders |
| Benign ovarian cyst |
| Menopausal disorders |
| Female infertility |
| Other specified female genital disorders |
| Postprocedural or postoperative genitourinary system complication |
| Tuberculosis |
| Septicemia |
| Bacterial infections |
| Fungal infections |
| Foodborne intoxications |
| HIV infection |
| Hepatitis |

| Viral infection |
| --- |
| Parasitic, other specified and unspecified infections |
| Sexually transmitted infections (excluding HIV and hepatitis) |
| Sequela of specified infectious disease conditions |
| Coronavirus disease – 2019 (COVID-19) |
| Fracture of head and neck, initial encounter |
| Fracture of the spine and back, initial encounter |
| Fracture of torso, initial encounter |
| Fracture of the upper limb, initial encounter |
| Fracture of the lower limb (except hip), initial encounter |
| Fracture of the neck of the femur (hip), initial encounter |
| Dislocations, initial encounter |
| Traumatic brain injury (TBI); concussion, initial encounter |
| Spinal cord injury (SCI), initial encounter |
| Internal organ injury, initial encounter |
| Open wounds of head and neck, initial encounter |
| Open wounds to limbs, initial encounter |
| Open wounds of trunk, initial encounter |
| Amputation of a limb, initial encounter |
| Amputation of other body parts, initial encounter |
| Injury to blood vessels, initial encounter |
| Superficial injury; contusion, initial encounter |
| Crushing injury, initial encounter |
| Burn and corrosion, initial encounter |
| Effect of foreign body entering opening, initial encounter |
| Effect of other external causes, initial encounter |
| Poisoning by drugs, initial encounter |
| Toxic effects, initial encounter |
| Sprains and strains, initial encounter |
| Injury to nerves, muscles and tendons, initial encounter |
| Other specified injury |
| Other unspecified injury |
| Adverse effects of drugs and medicaments, initial encounter |
| Underdosing of drugs and medicaments, initial encounter |
| Drug induced or toxic related condition |
| Allergic reactions |
| Maltreatment/abuse |
| Complication of cardiovascular device, implant or graft, initial encounter |
| Complication of genitourinary device, implant or graft, initial encounter |
| Complication of internal orthopedic device or implant, initial encounter |
| Complication of transplanted organs or tissue, initial encounter |
| Complication of other surgical or medical care, injury, initial encounter |
| Fracture of head and neck, subsequent encounter |
| Fracture of the spine and back, subsequent encounter |
| Fracture of torso, subsequent encounter |
| Fracture of the upper limb, subsequent encounter |
| Fracture of lower limb (except hip), subsequent encounter |
| Fracture of the neck of the femur (hip), subsequent encounter |
| Dislocations, subsequent encounter |

| Traumatic brain injury (TBI); concussion, subsequent encounter |
| --- |
| Spinal cord injury (SCI), subsequent encounter |
| Internal organ injury, subsequent encounter |
| Open wounds of head and neck, subsequent encounter |
| Open wounds to limbs, subsequent encounter |
| Open wounds of trunk, subsequent encounter |
| Amputation of a limb, subsequent encounter |
| Amputation of other body parts, subsequent encounter |
| Injury to blood vessels, subsequent encounter |
| Superficial injury; contusion, subsequent encounter |
| Crushing injury, subsequent encounter |
| Burns and corrosion, subsequent encounter |
| Effect of foreign body entering opening, subsequent encounter |
| Effect of other external causes, subsequent encounter |
| Poisoning by drugs, subsequent encounter |
| Toxic effects, subsequent encounter |
| Sprains and strains, subsequent encounter |
| Injury to nerves, muscles and tendons, subsequent encounter |
| Other specified injury, subsequent encounter |
| Other unspecified injuries, subsequent encounter |
| Adverse effects of drugs and medicaments, subsequent encounter |
| Underdosing of drugs and medicaments, subsequent encounter |
| Allergic reactions, subsequent encounter |
| Maltreatment/abuse, subsequent encounter |
| Complication of cardiovascular device, implant or graft, subsequent encounter |
| Complication of genitourinary device, implant or graft, subsequent encounter |
| Complication of internal orthopedic device or implant, subsequent encounter |
| Complication of other surgical or medical care, injury, subsequent encounter |
| Injury, sequela |
| Effect of other external causes, sequela |
| Poisoning/toxic effect/adverse effects/underdosing, sequela |
| Complication, sequela |
| Cardiac and circulatory congenital anomalies |
| Digestive congenital anomalies |
| Genitourinary congenital anomalies |
| Nervous system congenital anomalies |
| Congenital malformations of eye, ear, face, neck |
| Cleft lip or palate |
| Respiratory congenital malformations |
| Musculoskeletal congenital conditions |
| Chromosomal abnormalities |
| Other specified and unspecified congenital anomalies |
| Schizophrenia spectrum and other psychotic disorders |
| Depressive disorders |
| Bipolar and related disorders |
| Other specified and unspecified mood disorders |
| Anxiety and fear-related disorders |
| Obsessive-compulsive and related disorders |
| Trauma- and stressor-related disorders |

| Disruptive, impulse-control and conduct disorders |
| --- |
| Personality disorders |
| Feeding and eating disorders |
| Somatic disorders |
| Suicidal ideation/attempt/intentional self-harm |
| Miscellaneous mental and behavioral disorders/conditions |
| Neurodevelopmental disorders |
| Alcohol-related disorders |
| Opioid-related disorders |
| Cannabis-related disorders |
| Sedative-related disorders |
| Stimulant-related disorders |
| Hallucinogen-related disorders |
| Inhalant-related disorders |
| Tobacco-related disorders |
| Other specified substance-related disorders |
| Mental and substance use disorders in remission |
| Suicide attempt/intentional self-harm; subsequent encounter |
| Opioid-related disorders; subsequent encounter |
| Stimulant-related disorders; subsequent encounter |
| Cannabis-related disorders; subsequent encounter |
| Hallucinogen-related disorders; subsequent encounter |
| Sedative-related disorders; subsequent encounter |
| Inhalant-related disorders; subsequent encounter |
| Mental and substance use disorders; sequela |
| Infective arthritis |
| Osteomyelitis |
| Rheumatoid arthritis and related disease |
| Juvenile arthritis |
| Other specified chronic arthropathy |
| Osteoarthritis |
| Other specified joint disorders |
| Immune-mediated/reactive arthropathies |
| Tendon and synovial disorders |
| Musculoskeletal pain, not low back pain |
| Spondylopathies/spondyloarthropathy (including infective) |
| Biomechanical lesions |
| Osteoporosis |
| Pathological fracture, initial encounter |
| Pathological fracture, subsequent encounter |
| Stress fracture, initial encounter |
| Stress fracture, subsequent encounter |
| Atypical fracture, initial encounter |
| Atypical fracture, subsequent encounter |
| Pathological, stress and atypical fractures, sequela |
| Acquired foot deformities |
| Scoliosis and other postural dorsopathic deformities |
| Acquired deformities (excluding foot) |
| Systemic lupus erythematosus and connective tissue disorders |

| Other specified connective tissue disease |
| --- |
| Muscle disorders |
| Musculoskeletal abscess |
| Other specified bone disease and musculoskeletal deformities |
| Disorders of jaw |
| Aseptic necrosis and osteonecrosis |
| Traumatic arthropathy |
| Neurogenic/neuropathic arthropathy |
| Gout |
| Crystal arthropathies (excluding gout) |
| Osteomalacia |
| Autoinflammatory syndromes |
| Postprocedural or postoperative musculoskeletal system complication |
| Low back pain |
| Head and neck cancers - eye |
| Head and neck cancers - lip and oral cavity |
| Head and neck cancers - throat |
| Head and neck cancers - salivary gland |
| Head and neck cancers - nasopharyngeal |
| Head and neck cancers - hypopharyngeal |
| Head and neck cancers - pharyngeal |
| Head and neck cancers - laryngeal |
| Head and neck cancers - tonsils |
| Head and neck cancers - all other types |
| Cardiac cancers |
| Gastrointestinal cancers - esophagus |
| Gastrointestinal cancers - stomach |
| Gastrointestinal cancers - small intestine |
| Gastrointestinal cancers - colorectal |
| Gastrointestinal cancers - anus |
| Gastrointestinal cancers - liver |
| Gastrointestinal cancers - bile duct |
| Gastrointestinal cancers - gallbladder |
| Gastrointestinal cancers - peritoneum |
| Gastrointestinal cancers - all other types |
| Respiratory cancers |
| Bone cancer |
| Sarcoma |
| Skin cancers - melanoma |
| Skin cancers - basal cell carcinoma |
| Skin cancers - squamous cell carcinoma |
| Skin cancers - all other types |
| Breast cancer - ductal carcinoma in situ (DCIS) |
| Breast cancer - all other types |
| Female reproductive system cancers - uterus |
| Female reproductive system cancers - cervix |
| Female reproductive system cancers - ovary |
| Female reproductive system cancers - fallopian tube |
| Female reproductive system cancers - endometrium |

| Female reproductive system cancers - vulva |
| --- |
| Female reproductive system cancers - vagina |
| Female reproductive system cancers - all other types |
| Male reproductive system cancers - prostate |
| Male reproductive system cancers - testis |
| Male reproductive system cancers - penis |
| Male reproductive system cancers - all other types |
| Urinary system cancers - bladder |
| Urinary system cancers - ureter and renal pelvis |
| Urinary system cancers - kidney |
| Urinary system cancers - urethra |
| Urinary system cancers - all other types |
| Nervous system cancers - brain |
| Nervous system cancers - all other types |
| Endocrine system cancers - thyroid |
| Endocrine system cancers - pancreas |
| Endocrine system cancers - thymus |
| Endocrine system cancers - adrenocortical |
| Endocrine system cancers - parathyroid |
| Endocrine system cancers - pituitary gland |
| Endocrine system cancers - all other types |
| Hodgkin lymphoma |
| Non-Hodgkin lymphoma |
| Leukemia - acute lymphoblastic leukemia (ALL) |
| Leukemia - acute myeloid leukemia (AML) |
| Leukemia - chronic lymphocytic leukemia (CLL) |
| Leukemia - chronic myeloid leukemia (CML) |
| Leukemia - hairy cell |
| Leukemia - all other types |
| Multiple myeloma |
| Malignant neuroendocrine tumors |
| Mesothelioma |
| Myelodysplastic syndrome (MDS) |
| Cancer of other sites |
| Secondary malignancies |
| Malignant neoplasm, unspecified |
| Neoplasms of unspecified nature or uncertain behavior |
| Benign neoplasms |
| Conditions due to neoplasm or the treatment of neoplasm |
| Meningitis |
| Encephalitis |
| Other specified CNS infection and poliomyelitis |
| Parkinson`s disease |
| Multiple sclerosis |
| Other specified hereditary and degenerative nervous system conditions |
| Cerebral palsy |
| Paralysis (other than cerebral palsy) |
| Epilepsy; convulsions |
| Headache; including migraine |

| Neurocognitive disorders |
| --- |
| Transient cerebral ischemia |
| Coma; stupor; and brain damage |
| CNS abscess |
| Polyneuropathies |
| Sleep wake disorders |
| Nerve and nerve root disorders |
| Myopathies |
| Nervous system pain and pain syndromes |
| Other specified nervous system disorders |
| Postprocedural or postoperative nervous system complication |
| Sequela of specified nervous system conditions |
| Liveborn |
| Short gestation; low birth weight; and fetal growth retardation |
| Neonatal acidemia and hypoxia |
| Neonatal cerebral disorders |
| Respiratory distress syndrome |
| Respiratory perinatal condition |
| Hemolytic jaundice and perinatal jaundice |
| Birth trauma |
| Perinatal infections |
| Newborn affected by maternal conditions or complications of labor/delivery |
| Hemorrhagic and hematologic disorders of newborn |
| Neonatal digestive and feeding disorders |
| Other specified and unspecified perinatal conditions |
| Neonatal abstinence syndrome |
| Fetal alcohol syndrome |
| Antenatal screening |
| Gestational weeks |
| Spontaneous abortion and complications of spontaneous abortion |
| Induced abortion and complications of termination of pregnancy |
| Ectopic pregnancy and complications of ectopic pregnancy |
| Molar pregnancy and other abnormal products of conception |
| Complications following ectopic and/or molar pregnancy |
| Supervision of high-risk pregnancy |
| Early, first or unspecified trimester hemorrhage |
| Hemorrhage after first trimester |
| Early or threatened labor |
| Multiple gestation |
| Maternal care related to fetal conditions |
| Polyhydramnios and other problems of amniotic cavity |
| Obstetric history affecting care in pregnancy |
| Previous C-section |
| Maternal care for abnormality of pelvic organs |
| Maternal care related to disorders of the placenta and placental implantation |
| Diabetes or abnormal glucose tolerance complicating pregnancy; childbirth; or the puerperium |
| Hypertension and hypertensive-related conditions complicating pregnancy; childbirth; and the puerperium |
| Maternal intrauterine infection |

| Prolonged pregnancy |
| --- |
| Complications specified during childbirth |
| Malposition, disproportion or other labor complications |
| Anesthesia complications during pregnancy |
| OB-related trauma to perineum and vulva |
| Complications specified during the puerperium |
| Other specified complications in pregnancy |
| Uncomplicated pregnancy, delivery or puerperium |
| Maternal outcome of delivery |
| Sinusitis |
| Pneumonia (except that caused by tuberculosis) |
| Influenza |
| Acute and chronic tonsillitis |
| Acute bronchitis |
| Other specified upper respiratory infections |
| Other specified and unspecified upper respiratory disease |
| Chronic obstructive pulmonary disease and bronchiectasis |
| Asthma |
| Aspiration pneumonitis |
| Pleurisy, pleural effusion and pulmonary collapse |
| Respiratory failure; insufficiency; arrest |
| Lung disease due to external agents |
| Pneumothorax |
| Mediastinal disorders |
| Other specified and unspecified lower respiratory disease |
| Postprocedural or postoperative respiratory system complication |
| Skin and subcutaneous tissue infections |
| Other specified inflammatory condition of skin |
| Pressure ulcer of skin |
| Non-pressure ulcer of skin |
| Contact dermatitis |
| Postprocedural or postoperative skin complication |
| Other specified and unspecified skin disorders |
| Syncope |
| Fever |
| Shock |
| Nausea and vomiting |
| Dysphagia |
| Abdominal pain and other digestive/abdomen signs and symptoms |
| Malaise and fatigue |
| Symptoms of mental and substance use conditions |
| Abnormal findings related to substance use |
| Nervous system signs and symptoms |
| Genitourinary signs and symptoms |
| Circulatory signs and symptoms |
| Respiratory signs and symptoms |
| Skin/Subcutaneous signs and symptoms |
| General sensation/perception signs and symptoms |
| Other general signs and symptoms |

| Abnormal findings without diagnosis |
| --- |
| **CCSR Procedure Category Description (for details of ICD 10, refer to** [Clinical Classifications](https://www.hcup-us.ahrq.gov/toolssoftware/ccsr/ccs_refined.jsp)  [Software Refined (CCSR) (ahrq.gov)](https://www.hcup-us.ahrq.gov/toolssoftware/ccsr/ccs_refined.jsp) |
| Transfusion of blood and blood products |
| Transfusion of plasma |
| Administration of albumin and globulin |
| Transfusion of clotting factors |
| Administration of thrombolytics and platelet inhibitors |
| Infusion of vasopressor |
| Intravenous induction of labor |
| Cervical ripening |
| Rh immunoglobulin and other serum infusion |
| Vaccinations |
| Administration and transfusion of bone marrow, stem cells, pancreatic islet cells, and t-cells |
| Chemotherapy |
| Peritoneal dialysis |
| Regional anesthesia |
| Administration of antibiotics |
| Administration of anti-inflammatory agents |
| Administration of nutritional and electrolytic substances |
| Irrigation (diagnostic and therapeutic) |
| Potential COVID-19 therapies |
| Administration of diagnostic substances, NEC |
| Administration of therapeutic substances, NEC |
| Heart biopsy |
| Heart conduction mechanism procedures |
| Coronary artery bypass grafts (CABG) |
| Percutaneous coronary interventions (PCI) |
| Other coronary artery procedures (excluding CABG and PCI) |
| Carotid endarterectomy and stenting |
| Embolectomy, endarterectomy, and related vessel procedures (non-endovascular; excluding carotid) |
| Angioplasty and related vessel procedures (endovascular; excluding carotid) |
| Left atrial appendage procedures |
| Ligation and embolization of vessels |
| Aneurysm repair procedures |
| Vessel repair and replacement |
| Heart and great vessel bypass procedures |
| Peripheral arterial bypass procedures |
| Peripheral arteriovenous fistula and shunt procedures |
| Portal and other venous bypass procedures |
| Pericardial procedures |
| Heart transplant |
| Septal repair and other therapeutic heart procedures |
| Saphenous vein harvest and other therapeutic vessel removal |
| Artery, vein, and great vessel procedures, NEC |
| Heart valve replacement and other valve procedures (non-endovascular) |
| Heart valve replacement and other valve procedures (endovascular) |

| Venous and arterial catheter placement |
| --- |
| Placement of tunneled or implantable portion of a vascular access device |
| Pacemaker and defibrillator procedures |
| Heart assist device procedures |
| Inferior vena cava (IVC) filter procedures |
| Cardiovascular device procedures, NEC |
| Chiropractic manipulation |
| Minimally invasive CNS biopsy |
| Lumbar puncture |
| Spinal canal and spinal cord drainage (excluding lumbar puncture) |
| Intracranial epidural and subdural space drainage |
| Ventriculostomy [percutaneous approach] |
| Other CNS drainage and related procedures |
| CNS excision procedures |
| Spinal cord decompression |
| Decompressive craniectomy |
| Cerebrospinal fluid shunt procedures |
| Meninges repair |
| CNS neurostimulator procedures |
| Spinal epidural catheter placement |
| CNS procedures, NEC |
| Endocrine system biopsy |
| Thyroidectomy |
| Parathyroidectomy |
| Adrenalectomy |
| Pituitary gland resection |
| Endocrine procedures, NEC |
| Laryngoscopy (diagnostic) |
| ENT diagnostic endoscopy (excluding laryngoscopy) |
| ENT diagnostic procedures (non-endoscopic) |
| Diagnostic audiology |
| Myringotomy |
| ENT drainage (excluding myringotomy) |
| Tonsillectomy and adenoidectomy |
| Nasal and sinus excision |
| Tongue excision |
| Salivary gland excision |
| Laryngectomy |
| ENT excision (excluding nasal passage, sinuses, tongue, salivary glands, larynx) |
| ENT repair |
| Frenectomy |
| Dental procedures |
| Face transplant |
| ENT procedures, NEC |
| Hemodialysis |
| Extracorporeal membrane oxygenation |
| Mechanical ventilation |
| Non-invasive ventilation |
| Cardiac chest compression |

| Cardioversion |
| --- |
| Cardiac pacing |
| Open heart cardiopulmonary bypass |
| Cardiac assistance with balloon pump |
| Cardiac assistance with ventricular assist device |
| Hyperbaric oxygen therapy |
| Pheresis therapy |
| Phototherapy |
| Ultraviolet light therapy |
| Hypothermia therapy |
| Extracorporeal or systemic therapies, NEC |
| Eye procedures |
| Eyelid procedures |
| Hysterectomy |
| Oophorectomy |
| Salpingectomy |
| Trachelectomy |
| Fallopian tube ligation and excision |
| Vulvar laceration repair |
| Female genital tract repair (excluding vulva) |
| Cervical dilation |
| Cervical cerclage |
| Female upper genital tract excision |
| Female lower genital tract excision |
| Intrauterine device (IUD) insertion |
| Subcutaneous contraceptive implant |
| Ovary or uterus transplant |
| Female reproductive system procedures, NEC |
| Esophagogastroduodenoscopy (EGD) with biopsy |
| Colonoscopy and proctoscopy with biopsy |
| GI system biopsy (non-endoscopic) |
| GI system endoscopy without biopsy (diagnostic) |
| Exploration of peritoneal cavity |
| Paracentesis |
| GI system drainage (excluding paracentesis) |
| Appendectomy |
| Colectomy |
| Gastrectomy |
| Small bowel resection |
| Duodenal resection |
| Proctectomy or anal resection |
| Esophagectomy |
| Omentectomy or peritoneum resection |
| GI system endoscopic therapeutic procedures |
| Gastrostomy |
| Ileostomy and colostomy |
| Gastro-jejunal bypass (including bariatric) |
| Anorectal repair (3rd and 4th degree obstetrical repairs and other) |
| GI system repair (excluding anorectal) |

| GI system lysis of adhesions |
| --- |
| GI transplant |
| Other peritoneal cavity procedures |
| Other GI system device procedures |
| Upper GI therapeutic procedures, NEC (open and laparoscopic) |
| Upper GI therapeutic procedures, NEC (endoscopic) |
| Lower GI therapeutic procedures, NEC (open and laparoscopic) |
| Lower GI therapeutic procedures, NEC (excluding open and laparoscopic) |
| Cosmetic procedures |
| Abdominal wall repair (including hernia) |
| Inguinal and femoral hernia repair |
| Endoscopic control of bleeding |
| Control of bleeding (non-endoscopic) |
| Chest wall procedures, NEC |
| Mediastinal procedures, NEC |
| Abdominal wall procedures, NEC |
| Retroperitoneal procedures, NEC |
| Genitourinary tract procedures, NEC |
| Liver biopsy |
| Pancreaticobiliary biopsy |
| Diagnostic ERCP with or without biopsy |
| Hepatobiliary resection and ablation |
| Pancreatectomy |
| Cholecystectomy |
| Biliary and pancreatic calculus removal |
| Common bile duct sphincterotomy and stenting |
| Pancreatic and proximal biliary dilation and stenting |
| Hepatobiliary and pancreatic drainage |
| Liver transplant |
| Pancreas transplant |
| Hepatobiliary and pancreatic procedures, NEC |
| Cardiac and coronary fluoroscopy |
| Fluoroscopic angiography (excluding coronary) |
| Fluoroscopic guidance for circulatory system procedures |
| Fluoroscopy of non-circulatory organs |
| Magnetic resonance imaging (MRI) |
| Computerized tomography (CT) with contrast |
| Computerized tomography (CT) without contrast |
| Ultrasonography |
| Plain radiography |
| Fluorescence imaging |
| Lymph node biopsy |
| Lymph node excision (therapeutic) |
| Lymph node dissection |
| Thymectomy |
| Splenectomy |
| Spleen procedures (excluding splenectomy) |
| Bone marrow biopsy |
| Bone marrow aspiration (therapeutic) |

| Thymus transplant |
| --- |
| Spleen transplant |
| Lymphatic procedures, NEC |
| Arterial oxygen saturation monitoring |
| Peripheral arterial pressure monitoring |
| Pulmonary arterial pressure monitoring |
| Cardiac stress tests |
| Measurement during cardiac catheterization |
| Pacemaker and defibrillator interrogation |
| Electrocardiogram (ECG) |
| Cardiac monitoring |
| Electrophysiologic studies |
| Other cardiovascular system measurement and monitoring |
| Electroencephalogram (EEG) |
| ICP and other CNS monitoring (excluding EEG) |
| Monitoring of peripheral nerves |
| Pulmonary function tests |
| Measurement and monitoring, NEC |
| Pharmacotherapy for mental health (excluding substance use) |
| Psychotherapy for mental health (excluding substance use) |
| Electroconvulsive therapy |
| Crisis intervention for mental health |
| Mental health procedures, NEC |
| Circumcision |
| Penis procedures (excluding circumcision) |
| Prostatectomy |
| Prostate and seminal vesicle procedures (excluding prostatectomy) |
| Male perineum procedures |
| Male reproductive system transplant |
| Male reproductive system procedures, NEC |
| Arthrocentesis |
| Bone and joint biopsy |
| Subcutaneous tissue, fascia, and muscle biopsy |
| Incision and drainage of musculoskeletal tissue and joints |
| Incision and drainage of subcutaneous tissue and fascia |
| Knee arthroplasty |
| Hip arthroplasty |
| Arthroplasty of other joint (excluding knee and hip) |
| Fixation of upper extremity bones |
| Femur fixation |
| Fixation of leg and foot bones |
| Bone fixation (excluding extremities) |
| Spine fusion |
| Joint fusion (excluding spine) |
| Closed reduction of bones and joints |
| Vertebral discectomy |
| Joint tissue excision (excluding discectomy) |
| Bone excision |
| Muscle, tendon, bursa, and ligament excision |

| Subcutaneous tissue and fascia excision |
| --- |
| Perineal muscle laceration repair (2nd degree obstetrical and other) |
| Tendon, muscle, bursa, and ligament repair (excluding perineal) |
| Toe and mid foot amputation |
| Below knee amputation |
| Above knee and other proximal lower extremity amputation |
| Finger and other upper extremity amputation |
| Hand transplant |
| Subcutaneous tissue and fascia procedures, NEC |
| Musculoskeletal device procedures, NEC |
| Musculoskeletal procedures, NEC |
| Planar nuclear medicine imaging |
| Tomographic nuclear medicine imaging |
| Radionuclide therapy and non-imaging procedures |
| Positron emission tomographic (PET) imaging |
| Osteopathic treatment |
| Robotic-assisted procedures |
| Computer-assisted procedures |
| Fluorescence-guided procedures |
| Isolation procedures |
| Therapeutic massage and related procedures |
| Fetal heart rate monitoring |
| Spontaneous vaginal delivery |
| Cesarean section |
| Assisted vaginal delivery |
| Episiotomy |
| Removal of placenta and other retained products of conception |
| Removal of ectopic pregnancy |
| Abortion |
| Pregnancy and fetal procedures, NEC |
| Immobilization by splint or other external device |
| Packing and dressing procedures |
| Lumbosacral nerve decompression |
| Cervicothoracic nerve decompression |
| Peripheral nerve decompression (excluding lumbosacral and cervicothoracic) |
| Nerve repair |
| Peripheral nerve denervation |
| Peripheral nerve procedures, NEC |
| Beam radiation |
| Brachytherapy |
| Stereotactic radiosurgery |
| Radiation therapy, NEC |
| Bronchoscopy (diagnostic) |
| Lung, pleura, or diaphragm biopsy (non-endoscopic) |
| Thoracentesis (diagnostic) |
| Bronchoscopy (therapeutic) |
| Chest tube placement and therapeutic thoracentesis |
| Open and thoracoscopic pleural drainage |
| Airway intubation |

| Lung, pleura, or diaphragm resection (open and thoracoscopic) |
| --- |
| Bronchoscopic excision and fulguration |
| Tracheostomy |
| Diaphragmatic hernia repair |
| Release of lung and pleura |
| Lung transplant |
| Respiratory system procedures, NEC |
| Physical, occupational, and respiratory therapy evaluation |
| Physical, occupational, and respiratory therapy treatment |
| Speech therapy evaluation |
| Speech therapy treatment |
| Skin biopsy and diagnostic drainage |
| Incision and drainage of skin |
| Mastectomy and lumpectomy |
| Breast reconstruction |
| Perineal skin repair (1st degree obstetrical and other) |
| Skin laceration repair (excluding perineum) |
| Skin graft |
| Skin excision and debridement |
| Nail procedures |
| Skin and breast procedures, NEC |
| Substance use detoxification |
| Pharmacotherapy for substance use |
| Psychotherapy for substance use |
| Counseling for substance use |
| Cystoscopy and ureteroscopy (including biopsy) |
| Kidney and other urinary tract biopsy (non-endoscopic) |
| Ureter and other urinary tract dilation |
| Removal of calculi and other matter from urinary tract |
| Nephrostomy and ureterostomy procedures (including stents) |
| Bladder catheterization and drainage |
| Urinary tract repair |
| Nephrectomy and ureterectomy |
| Cystectomy (including fulguration) and urethrectomy |
| Urinary diversion with anastomosis or ostomy |
| Kidney transplant |
| Urinary system procedures, NEC |

# Supplemental Table 1:

All candidate diagnosis and procedural codes that were initially considered

in building the TAVI readmission score

| **Disease/ Complication** | **ICD-10-CM** |
| --- | --- |
| Adverse drug events | T36**, T37**, T38**, T39**, T40**, T41**, T42**, T43**, T44**, T45**, T46**, T47**, T48**, T49**, T50** |
| COPD | J449, J441 |
| CVA (ischemic or hemorrhagic) | I60**, I61**, I62**, I63**, I60**, I61**, I62**, I63**, 430**, 431**, 432**, 43301, 43331, 43381, 43401, 43411, 430**, 431**, 432**, 43301, 43331, 43381,  43401, 43411, 43491 |
| Elixhauser comorbidities | We used the Healthcare Cost and Utilization Project (HCUP) Elixhauser comorbidity software (beta version 2020.1). |
| Frailty score | Please refer to Gilbert, T., et al. (2018). "Development and validation of a Hospital Frailty Risk Score focusing on older people in acute care settings using electronic hospital records: an observational study." Lancet **391**(10132): 1775-1782. |
| GI bleeding | I8501, I8511, K250, K252, K254, K256, K260, K262, K264, K266, K270, K272, K274, K276, K280, K282, K284, K286, K625, K920, K921, K922 |
| HCUP Clinical Classification Software (CCS ) diagnoses | 542 variables. We used the CCSR v2021.1 version. Please refer to [Tools Archive](https://www.hcup-us.ahrq.gov/toolssoftware/ccsr/ccsr_archive.jsp#ccsr) [for Clinical Classifications Software Refined (ahrq.gov)](https://www.hcup-us.ahrq.gov/toolssoftware/ccsr/ccsr_archive.jsp#ccsr) |
| HCUP Clinical Classification Software (CCS ) procedures | 326 variables. We used the CCSR v2021.1. Please refer to [Tools Archive for](https://www.hcup-us.ahrq.gov/toolssoftware/ccsr/ccsr_archive.jsp#ccspcs) [Clinical Classifications Software Refined (ahrq.gov)](https://www.hcup-us.ahrq.gov/toolssoftware/ccsr/ccsr_archive.jsp#ccspcs) |
| Hyponatremia | E87.1 |
| Myocardial infarction | I21** |
| Transcatheter aortic valve implantation | 02RF37H, 02RF37Z, 02RF38H, 02RF38Z, 02RF3JH, 02RF3JZ, 02RF3KH,  02RF3KZ |
| Pneumonia | J95851, J954, A0103, A0222, A202, A212, A221, A310, A3701, A3711, A430, A481, B012, B052, B0681, B250, B371, B380, B381, B382, B390, B391, B392, B583, B59, B7781, J120, J121, J122, J123, J1281, J1289, J129, J13, J14, J150, J151, J1520, J15211, J15212, J1529, J153, J154, J155, J156, J157, J158, J159, J160, J168, J17, J180, J181, J188, J189, J851. |
| Septicemia | A021, A207, A227, A267, A327, A392, A393, A394, A400, A401, A403, A408, A409, A4101, A4102, A411, A412, A413, A414, A4150, A4151, A4152, A4153, A4159, A4181, A4189, A419, A427, A5486, B007, B377, I76, P360, P3610, P3619, P362, P3630, P3639, P364, P365, P368, P369, R6520 |
| Therapeutic errors | Y62**, Y63**, Y64**, Y65**, Y66**, Y69** |

# Supplemental Table 2:

International Classification of Diseases-Clinical Modifications (ICD- CM10) Codes that were used in our study

| **Rank** | **Predictor** | **Coefficient** |
| --- | --- | --- |
| 1 | Preadmitted within 30 days | 0.13 |
| 2 | Cardiac dysrhythmias | 0.11 |
| 3 | Chronic obstructive pulmonary disease and bronchiectasis | 0.08 |
| 4 | Discharge to a skilled nursing facility | 0.079 |
| 5 | Renal failure (Elixhauser comorbidity) | 0.06 |
| 6 | Hemodialysis | 0.058 |
| 7 | TAVR volume | -0.056 |
| 8 | Length of stay | 0.054 |
| 9 | Deficiency anemia (Elixhauser comorbidity) | 0.053 |
| 10 | Transfusion of blood and blood products | 0.051 |
| 11 | Complication of cardiovascular device, implant or graft, initial encounter | -0.049 |
| 12 | AG | 0.049 |
| 13 | Frailty score | 0.045 |
| 14 | Conduction disorders | 0.044 |
| 15 | Number of diagnoses | 0.041 |
| 16 | Diabetes mellitus with complication | 0.039 |
| 17 | Heart failure | 0.037 |
| 18 | Respiratory failure; insufficiency; arrest | 0.036 |
| 19 | Acute and unspecified renal failure | 0.034 |
| 20 | ELECTIVE | -0.034 |
| 21 | Hyponatremia | 0.032 |
| 22 | Elixhauser score | 0.032 |
| 23 | Embolectomy, endarterectomy, and related vessel procedures (non-endovascular; excluding carotid) | 0.031 |
| 24 | Disorders of lipid metabolism | -0.03 |
| 25 | Vessel repair and replacement | 0.029 |
| 26 | Postprocedural or postoperative respiratory system complication | 0.024 |
| 27 | Patient Location: NCHS Urban-Rural Code (ranging from 1 "Central" counties of metro areas of >=1 million population to 6 "Not metropolitan") | -0.024 |
| 28 | Medicare/aid insurance | 0.022 |
| 29 | Other specified male genital disorders | 0.021 |
| 30 | Pericarditis and pericardial disease | 0.021 |
| 31 | Myelodysplastic syndrome (MDS) | 0.02 |
| 32 | Genitourinary signs and symptoms (for detailed ICD-10 list, see HCUP CCS [Research Tools (ahrq.gov)](https://www.hcup-us.ahrq.gov/tools_software.jsp) | 0.019 |
| 33 | Angioplasty and related vessel procedures (endovascular; excluding carotid) | 0.019 |
| 34 | Hematuria | 0.018 |
| 35 | Peritoneal dialysis | 0.018 |
| 36 | Musculoskeletal device procedures, NEC (for detailed ICD-10 list, see HCUP CCS [Research Tools (ahrq.gov)](https://www.hcup-us.ahrq.gov/tools_software.jsp) | 0.017 |

| 37 | Pulmonary heart disease | 0.015 |
| --- | --- | --- |
| 38 | Hodgkin lymphoma | 0.015 |
| 39 | Bone fixation (excluding extremities) | 0.014 |
| 40 | Other specified and unspecified diseases of kidney and ureters (for detailed ICD- 10 list, see HCUP CCS [Research Tools (ahrq.gov)](https://www.hcup-us.ahrq.gov/tools_software.jsp) | 0.014 |
| 41 | Non-pressure ulcer of skin | 0.014 |
| 42 | Tracheostomy | -0.014 |
| 43 | Complication of transplanted organs or tissue, initial encounter (for detailed ICD-  10 list, see HCUP CCS [Research Tools (ahrq.gov)](https://www.hcup-us.ahrq.gov/tools_software.jsp) | 0.013 |
| 44 | Female reproductive system cancers - endometrium | 0.013 |
| 45 | Acute myocardial infarction | -0.013 |
| 46 | Anal and rectal conditions | 0.013 |
| 47 | Peripheral arterial bypass procedures | 0.013 |
| 48 | Hypertension (Elixhauser comorbidity) | -0.012 |
| 49 | Fluid and electrolyte disorders (for detailed ICD-10 list, see HCUP CCS [Research](https://www.hcup-us.ahrq.gov/tools_software.jsp) [Tools (ahrq.gov)](https://www.hcup-us.ahrq.gov/tools_software.jsp) | 0.012 |
| 50 | Leukemia - acute myeloid leukemia (AML) | 0.012 |
| 51 | Sleep wake disorders | 0.011 |
| 52 | Placement of tunneled or implantable portion of a vascular access device | -0.011 |
| 53 | Noninfectious hepatitis | 0.011 |
| 54 | Hepatobiliary and pancreatic drainage | 0.01 |
| 55 | Amputation of a limb, initial encounter | 0.01 |
| 56 | Hypertension with complications and secondary hypertension | 0.009 |
| 57 | Respiratory cancers | 0.009 |
| 58 | Male reproductive system procedures, NEC | 0.009 |
| 59 | Depression (Elixhauser comorbidities) | 0.009 |
| 60 | Pericardial procedures | 0.009 |
| 61 | Cardiac chest compression | -0.008 |
| 62 | Incision and drainage of musculoskeletal tissue and joints | 0.008 |
| 63 | Other specified upper respiratory infections (for detailed ICD-10 list, see HCUP CCS [Research Tools (ahrq.gov)](https://www.hcup-us.ahrq.gov/tools_software.jsp) | -0.008 |
| 64 | Skin biopsy and diagnostic drainage | -0.007 |
| 65 | Closed reduction of bones and joints | 0.007 |
| 66 | Portal and other venous bypass procedures | 0.007 |
| 67 | Nervous system pain and pain syndromes | 0.007 |
| 68 | Malignant neoplasm, unspecified | 0.007 |
| 69 | Urinary incontinence | -0.007 |
| 70 | Immune-mediated/reactive arthropathies | 0.006 |
| 71 | Diseases of white blood cells | 0.006 |
| 72 | Chronic rheumatic heart disease | 0.006 |

# Supplemental Table 3:

# Informative predictors for 30-day readmission by lasso method

| **Rank** | **Predictor** | **Coefficient** |
| --- | --- | --- |
| 1 | Cardiac dysrhythmias | 0.1416 |
| 2 | Preadmitted withing 30 days | 0.1329 |
| 3 | Discharge to skilled nursing facility | 0.1127 |
| 4 | Renal failure (Elixhauser comorbidity) | 0.0682 |
| 5 | Total number of diagnoses | 0.0660 |
| 6 | Chronic obstructive pulmonary disease and bronchiectasis | 0.0654 |
| 7 | Diabetes with chronic complications (Elixhauser comorbidity) | 0.0633 |
| 8 | Deficiency anemia (Elixhauser comorbidity) | 0.0612 |
| 9 | Hemodialysis | 0.0569 |
| 10 | LOS (length of stay) | 0.0550 |
| 11 | TAVR hospital volume | -0.0528 |
| 12 | Transfusion of blood and blood products | 0.0491 |
| 13 | Heart failure | 0.0479 |
| 14 | Complication of cardiovascular device, implant or graft, initial encounter | -0.0477 |
| 15 | Elixhauser score | 0.0475 |
| 16 | Solid tumor (Elixhauser comorbidity) | 0.0440 |
| 17 | Disorders of lipid metabolism | -0.0427 |
| 18 | Frailty score | 0.0343 |
| 19 | Patient Location: NCHS Urban-Rural Code (ranging from 1 "Central" counties of metro areas of >=1 million population to 6 "Not metropolitan or counties") | -0.0325 |
| 20 | Respiratory failure; insufficiency; arrest | 0.0312 |
| 21 | Age | 0.0307 |
| 22 | Complication of transplanted organs or tissue, initial encounter | 0.0300 |
| 23 | Acute and unspecified renal failure | 0.0256 |
| 24 | Gastrointestinal cancers - colorectal | 0.0226 |
| 25 | Respiratory cancers | 0.0211 |
| 26 | Myelodysplastic syndrome (MDS) | 0.0208 |
| 27 | Elective admission | -0.0206 |
| 28 | Breast cancer - all other types | -0.0202 |
| 29 | Hyponatremia | 0.0202 |
| 30 | Cerebral infarction | -0.0198 |
| 31 | Female reproductive system cancers - endometrium | 0.0186 |
| 32 | Pericarditis and pericardial disease | 0.0185 |
| 33 | Embolectomy, endarterectomy, and related vessel procedures (non- endovascular; excluding carotid) | 0.0182 |
| 34 | Medicare/aid insurance | 0.0164 |
| 35 | Genitourinary signs and symptoms | 0.0163 |
| 36 | Cardiac and circulatory congenital anomalies | -0.0162 |
| 37 | Angioplasty and related vessel procedures (endovascular; excluding carotid) | 0.0152 |
| 38 | Skin and subcutaneous tissue infections | 0.0147 |

| 39 | Aortic; peripheral; and visceral artery aneurysms | 0.0144 |
| --- | --- | --- |
| 40 | Gastritis and duodenitis | 0.0131 |
| 41 | Mesothelioma | 0.0125 |
| 42 | Chronic rheumatic heart disease | 0.0124 |
| 43 | Peritoneal dialysis | 0.0124 |
| 44 | Depression (Elixhuaser comorbidity) | 0.0123 |
| 45 | Male reproductive system cancers - prostate | -0.0121 |
| 46 | Hypertension with complications and secondary hypertension | 0.0119 |
| 47 | Postprocedural or postoperative respiratory system complication | 0.0115 |
| 48 | Pulmonary heart disease | 0.0111 |
| 49 | Shock | -0.0110 |
| 50 | Pulmonary circulation disease (Elixhuaser comorbidity) | -0.0110 |
| 51 | Lymphoma (Elixhuaser comorbidity) | 0.0106 |
| 52 | Acute myocardial infarction | -0.0106 |
| 53 | Urinary tract infections | 0.0104 |
| 54 | Systemic lupus erythematosus and connective tissue disorders | 0.0101 |
| 55 | Hematuria | 0.0098 |
| 56 | Female gender | 0.0095 |
| 57 | Noninfectious hepatitis | 0.0094 |
| 58 | Paracentesis | 0.0091 |
| 59 | Extracorporeal membrane oxygenation | -0.0090 |
| 60 | Closed reduction of bones and joints | 0.0088 |
| 61 | Endocrine system cancers - pancreas | 0.0088 |
| 62 | Immune-mediated/reactive arthropathies | 0.0088 |
| 63 | Stress fracture, initial encounter | 0.0085 |
| 64 | Musculoskeletal device procedures, NEC | 0.0084 |
| 65 | Venous and arterial catheter placement | -0.0083 |
| 66 | Vessel repair and replacement | 0.0083 |
| 67 | Gastro-jejunal bypass (including bariatric) | 0.0082 |
| 68 | Inflammatory diseases of female pelvic organs | 0.0081 |
| 69 | Other peritoneal cavity procedures | 0.0081 |
| 70 | Leukemia - acute myeloid leukemia (AML) | 0.0081 |
| 71 | Uveitis and ocular inflammation | -0.0081 |
| 72 | Opioid-related disorders | 0.0081 |
| 73 | Parkinson`s disease | 0.0078 |
| 74 | Pneumothorax | 0.0077 |
| 75 | Other specified and unspecified diseases of kidney and ureters | 0.0076 |
| 76 | Leukemia - acute lymphoblastic leukemia (ALL) | 0.0073 |
| 77 | Skin biopsy and diagnostic drainage | -0.0070 |
| 78 | CHF (Elixhuaser comorbidity) | -0.0069 |
| 79 | Fracture of torso, subsequent encounter | 0.0067 |

| 80 | Leukemia - chronic myeloid leukemia (CML) | 0.0067 |
| --- | --- | --- |
| 81 | Computer-assisted procedures | -0.0066 |
| 82 | Septicemia | -0.0066 |
| 83 | Postprocedural or postoperative digestive system complication | 0.0066 |
| 84 | Trauma- and stressor-related disorders | -0.0066 |
| 85 | Fluoroscopy of non-circulatory organs | 0.0066 |
| 86 | Other specified complications in pregnancy | 0.0066 |
| 87 | Bone marrow biopsy | 0.0065 |
| 88 | Pressure ulcer of skin | 0.0065 |
| 89 | GI system repair (excluding anorectal) | -0.0064 |
| 90 | Rheumatoid arthritis and related disease | 0.0064 |
| 91 | Pacemaker and defibrillator interrogation | 0.0062 |
| 92 | Osteoporosis | -0.0061 |
| 93 | Fracture of the neck of the femur (hip), initial encounter | 0.0061 |
| 94 | Head and neck cancers - tonsils | 0.0061 |
| 95 | Other specified upper respiratory infections | -0.0058 |
| 96 | Peritonitis and intra-abdominal abscess | -0.0057 |
| 97 | Physical, occupational, and respiratory therapy evaluation | -0.0056 |
| 98 | Amputation of a limb, initial encounter | 0.0054 |
| 99 | Placement of tunneled or implantable portion of a vascular access device | -0.0054 |
| 100 | Other specified diseases of veins and lymphatics | 0.0054 |
| 101 | Gastrointestinal cancers - peritoneum | 0.0053 |
| 102 | Influenza | -0.0053 |
| 103 | Diseases of mouth; excluding dental | 0.0052 |
| 104 | Head and neck cancers - throat | 0.0052 |
| 105 | Anal and rectal conditions | 0.0052 |
| 106 | Lung disease due to external agents | 0.0050 |
| 107 | Other specified and unspecified liver disease | 0.0048 |
| 108 | Positron emission tomographic (PET) imaging | 0.0047 |
| 109 | Erectile dysfunction | -0.0047 |
| 110 | Proteinuria | -0.0046 |
| 111 | Disorders of jaw | 0.0045 |
| 112 | Incision and drainage of musculoskeletal tissue and joints | 0.0044 |
| 113 | Prostate and seminal vesicle procedures (excluding prostatectomy) | 0.0044 |
| 114 | Peripheral arterial bypass procedures | 0.0043 |
| 115 | Bronchoscopy (diagnostic) | -0.0043 |
| 116 | Teaching Hospital | -0.0042 |
| 117 | Eye procedures | 0.0042 |
| 118 | Weight loss (Elixhuaser comorbidity) | 0.0042 |
| 119 | Planar nuclear medicine imaging | -0.0040 |
| 120 | Pathological, stress and atypical fractures, sequela | 0.0040 |

| 121 | Crystal arthropathies (excluding gout) | 0.0040 |
| --- | --- | --- |
| 122 | Sequela of hemorrhagic cerebrovascular disease | -0.0039 |
| 123 | Other specified male genital disorders | 0.0039 |
| 124 | Metastatic cancer (Elixhuaser comorbidity) | 0.0038 |
| 125 | Injury to nerves, muscles and tendons, initial encounter | 0.0036 |
| 126 | Removal of calculi and other matter from urinary tract | 0.0036 |
| 127 | Other specified and unspecified gastrointestinal disorders | 0.0035 |
| 128 | Genitourinary congenital anomalies | 0.0035 |
| 129 | Abnormal findings without diagnosis | -0.0034 |
| 130 | Code is unacceptable as a principal diagnosis PDX (only used for the inpatient default CCSR) | -0.0034 |
| 131 | Diseases of white blood cells | 0.0034 |
| 132 | Infusion of vasopressor | -0.0032 |
| 133 | Fracture of head and neck, subsequent encounter | -0.0031 |
| 134 | Urinary system cancers - ureter and renal pelvis | 0.0030 |
| 135 | Diabetes mellitus (Elixhuaser comorbidity) | 0.0029 |
| 136 | Abdominal pain and other digestive/abdomen signs and symptoms | 0.0029 |
| 137 | Head and neck cancers - laryngeal | 0.0029 |
| 138 | Suicidal ideation/attempt/intentional self-harm | 0.0028 |
| 139 | Fixation of upper extremity bones | 0.0028 |
| 140 | Transfusion of clotting factors | -0.0028 |
| 141 | Open wounds to limbs, subsequent encounter | 0.0028 |
| 142 | Breast cancer - ductal carcinoma in situ (DCIS) | 0.0028 |
| 143 | Gastrectomy | 0.0027 |
| 144 | Open wounds to limbs, initial encounter | 0.0027 |
| 145 | Circulatory signs and symptoms | -0.0027 |
| 146 | Obsessive-compulsive and related disorders | 0.0026 |
| 147 | Complication, sequela | -0.0026 |
| 148 | Sarcoma | 0.0025 |
| 149 | Coagulopathy (Elixhuaser comorbidity) | -0.0025 |
| 150 | Encephalitis | -0.0025 |
| 151 | Cerebral palsy | -0.0024 |
| 152 | Conduction disorders | 0.0024 |
| 153 | Vertebral discectomy | -0.0023 |
| 154 | Multiple myeloma | 0.0023 |
| 155 | Hypertension (Elixhuaser comorbidity) | -0.0022 |
| 156 | Urinary system cancers - kidney | 0.0022 |
| 157 | Fracture of head and neck, initial encounter | -0.0021 |
| 158 | Hepatobiliary and pancreatic drainage | 0.0021 |
| 159 | Gastrointestinal cancers - bile duct | 0.0021 |
| 160 | Bone fixation (excluding extremities) | 0.0020 |

| 161 | Osteopathic treatment | 0.0018 |
| --- | --- | --- |
| 162 | Administration of diagnostic substances, NEC | -0.0018 |
| 163 | Spinal epidural catheter placement | -0.0018 |
| 164 | Nonrheumatic and unspecified valve disorders | -0.0018 |
| 165 | Cardiac pacing | 0.0017 |
| 166 | Coma; stupor; and brain damage | -0.0017 |
| 167 | Musculoskeletal pain, not low back pain | 0.0017 |
| 168 | Tracheostomy | -0.0016 |
| 169 | Pericardial procedures | 0.0016 |
| 170 | Percutaneous coronary interventions (PCI) | -0.0014 |
| 171 | Lower GI therapeutic procedures, NEC (excluding open and laparoscopic) | -0.0014 |
| 172 | Adverse effects of drugs and medicaments, initial encounter | 0.0013 |
| 173 | Polyneuropathies | 0.0013 |
| 174 | Other specified substance-related disorders | -0.0013 |
| 175 | Postprocedural or postoperative complications of the spleen | 0.0011 |
| 176 | Therapeutic error | 0.0011 |
| 177 | Retroperitoneal procedures, NEC | 0.0010 |
| 178 | ENT excision (excluding nasal passage, sinuses, tongue, salivary glands,  larynx) | -0.0010 |
| 179 | Heart valve replacement and other valve procedures (non-endovascular) | -0.0010 |
| 180 | Extracorporeal or systemic therapies, NEC | 0.0009 |
| 181 | Spleen procedures (excluding splenectomy) | 0.0009 |
| 182 | Medical examination/evaluation | -0.0009 |
| 183 | Drug induced or toxic related condition | 0.0008 |
| 184 | Hodgkin lymphoma | 0.0007 |
| 185 | Cancer of other sites | 0.0007 |
| 186 | Skin cancers - basal cell carcinoma | -0.0007 |
| 187 | Colectomy | -0.0006 |
| 188 | Other coronary artery procedures (excluding CABG and PCI) | -0.0005 |
| 189 | Gastrostomy | -0.0005 |
| 190 | Sickle cell trait/anemia | 0.0004 |
| 191 | Nervous system pain and pain syndromes | 0.0004 |
| 192 | Asthma | -0.0002 |
| 193 | ENT diagnostic endoscopy (excluding laryngoscopy) | 0.0002 |
| 194 | Other specified and unspecified mood disorders | -0.0002 |
| 195 | Gastrointestinal and biliary perforation | -0.0002 |
| 196 | Effect of foreign body entering opening, initial encounter | 0.0001 |
| 197 | Female reproductive system cancers - ovary | 0.0001 |
| 198 | Carotid endarterectomy and stenting | <0.0001 |
| 199 | Pleurisy, pleural effusion, and pulmonary collapse | <0.0001 |

**Supplemental Table 4:**

Informative predictors for 90-day readmission by lasso method

| **Baseline Characteristics** | **30-day development cohort**  **n= 117,398** | **30-day validation cohort n=976** | **Absolute**  **SD** |
| --- | --- | --- | --- |
| **Demographics & hospital characteristics** |  |  |  |
| Age (mean+/-SD), years | 79.5 (8.4) | 77.9 (8.8) | 0.19 |
| Female sex | 45.2% | 40.9% | 0.09 |
| Lowest quartile household income | 19.4% | 4.7% | 0.46 |
| Medicare/Medicaid insurance | 91.5% | 88.4% | 0.10 |
| Hospital TAVR volume median (IQR) | 131(79-233) | 145(112-201) | 0.12 |
| Urban location | 93.5% | 98.0% | 0.19 |
| **Clinical Risk Factors** |  |  |  |
| ***Cardiovascular co-morbidities*** |  |  |  |
| Arrhythmia* | 51.2% | 55.1% | 0.22 |
| Congestive heart failure* | 0.9% | 0.9% | 0.08 |
| CVA (ischemic or hemorrhagic) | 1.7% | 1.8% | 0.01 |
| Diabetes without chronic complications* | 15.8% | 15.0% | 0.01 |
| Diabetes with chronic complications* | 21.7% | 22.5% | 0.02 |
| Obesity* | 19.6% | 24.9% | 0.99 |
| Peripheral vascular disease* | 22.1% | 17.8% | 0.13 |
| Valvular disease* | 1.0% | 1.4% | 0.11 |
| ***Non-cardiovascular comorbidities*** |  |  |  |
| Blood loss anemia* | 1.0% | 1.9% | 0.07 |
| Chronic pulmonary disease* | 27.8% | 24.0% | 0.09 |
| Coagulation deficiency* | 11.6% | 9.8% | 0.06 |
| Deficiency anemias* | 20.5% | 23.8% | 0.08 |
| Liver disease* | 3.1% | 4.0% | 0.05 |
| **Predictors** |  |  |  |
| Length of stay  Median (IQR) | 2.0(1.0-4.0) | 2.0(1.0-4.0) | 0.27 |
| Frailty score  Median (IQR) | 2.9(1.5-5.3) | 3.0(1.5-5.7) | 0.08 |
| Number of diagnoses Median (IQR) | 16.0(12.0-20.0) | 18.0(14.0-24.0) | 0.33 |
| Acute Kidney Injury | 9.7% | 9.2% | 0.02 |
| Elixhauser score*  Median (IQR) | 3.0(0.0-7.0) | 2.0(0.0-6.0) | 0.15 |
| **30-day readmission rate** | 12.4% | 12.0% | 0.01 |

**Supplemental Table 5**

Comparison of key baseline characteristics, predictors, and 30-day readmission rates between the development (NRD 2016-2019) and the validation data (Maryland SID 2020)

*****Defined by HCUP Elixhauser comorbidities software

IQR; interquartile range, N; number, SD; standardized difference.

| **Section/Topic** | **Item** |  | **Checklist Item** | **Page** |
| --- | --- | --- | --- | --- |
| **Title and abstract** | | | | |
| Title | 1 | D;V | Identify the study as developing and/or validating a multivariable prediction model, the target population, and the outcome to be predicted. | **1** |
| Abstract | 2 | D;V | Provide a summary of objectives, study design, setting, participants, sample size, predictors, outcome, statistical analysis, results, and conclusions. | **2** |
| **Introduction** | | | | |
| Background and objectives | 3a | D;V | Explain the medical context (including whether diagnostic or prognostic) and rationale for developing or validating the multivariable prediction model, including references to existing models. | **4** |
|  | 3b | D;V | Specify the objectives, including whether the study describes the development or validation of the model or both. | **4** |
| **Methods** | | | | |
| Source of data | 4a | D;V | Describe the study design or source of data (e.g., randomized trial, cohort, or registry data), separately for the development and validation data sets, if applicable. | **4** |
|  | 4b | D;V | Specify the key study dates, including start of accrual; end of accrual; and, if applicable, end of follow-up. | **4-5** |
| Participants | 5a | D;V | Specify key elements of the study setting (e.g., primary care, secondary care, general population) including number and location of centres. | **4-5** |
|  | 5b | D;V | Describe eligibility criteria for participants. | **5** |
|  | 5c | D;V | Give details of treatments received, if relevant. | **NA** |
| Outcome | 6a | D;V | Clearly define the outcome that is predicted by the prediction model, including how and when assessed. | **5** |
|  | 6b | D;V | Report any actions to blind assessment of the outcome to be predicted. | **NA** |
| Predictors | 7a | D;V | Clearly define all predictors used in developing or validating the multivariable prediction model, including how and when they were measured. | **5** |
|  | 7b | D;V | Report any actions to blind assessment of predictors for the outcome and other predictors. | **NA** |
| Sample size | 8 | D;V | Explain how the study size was arrived at. | **4-5** |
| Missing data | 9 | D;V | Describe how missing data were handled (e.g., complete-case analysis, single imputation, multiple imputation) with details of any imputation method. | **6** |
| Statistical analysis methods | 10a | D | Describe how predictors were handled in the analyses. | **5-6** |
|  | 10b | D | Specify type of model, all model-building procedures (including any predictor selection), and method for internal validation. | **5-6** |
|  | 10c | V | For validation, describe how the predictions were calculated. | **6** |
|  | 10d | D;V | Specify all measures used to assess model performance and, if relevant, to compare multiple models. | **8** |
|  | 10e | V | Describe any model updating (e.g., recalibration) arising from the validation, if done. | **NA** |
| Risk groups | 11 | D;V | Provide details on how risk groups were created, if done. | **7-8** |
| Development vs. validation | 12 | V | For validation, identify any differences from the development data in setting, eligibility criteria, outcome, and predictors. | **NA** |
| **Results** | | | | |
| Participants | 13a | D;V | Describe the flow of participants through the study, including the number of participants with and without the outcome and, if applicable, a summary of the follow-up time. A diagram may be helpful. | **6 and Fig 1** |
|  | 13b | D;V | Describe the characteristics of the participants (basic demographics, clinical features, available predictors), including the number of participants with missing data for predictors and outcome. | **7** |
|  | 13c | V | For validation, show a comparison with the development data of the distribution of important variables (demographics, predictors and outcome). | **eTable-5** |
| Model development | 14a | D | Specify the number of participants and outcome events in each analysis. | **6** |
|  | 14b | D | If done, report the unadjusted association between each candidate predictor and outcome. | **NA** |
| Model specification | 15a | D | Present the full prediction model to allow predictions for individuals (i.e., all regression coefficients, and model intercept or baseline survival at a given time point). | **8** |
|  | 15b | D | Explain how to the use the prediction model. | **8-9** |
| Model performance | 16 | D;V | Report performance measures (with CIs) for the prediction model. | **8** |
| Model-updating | 17 | V | If done, report the results from any model updating (i.e., model specification, model performance). | **na** |
| **Discussion** | | | | |
| Limitations | 18 | D;V | Discuss any limitations of the study (such as nonrepresentative sample, few events per predictor, missing data). | **11** |
| Interpretation | 19a | V | For validation, discuss the results with reference to performance in the development data, and any other validation data. | **8** |
|  | 19b | D;V | Give an overall interpretation of the results, considering objectives, limitations, results from similar studies, and other relevant evidence. | **11** |
| Implications | 20 | D;V | Discuss the potential clinical use of the model and implications for future research. | **10** |
| **Other information** | | | | |
| Supplementary information | 21 | D;V | Provide information about the availability of supplementary resources, such as study protocol, Web calculator, and data sets. | **Suppl matrial** |
| Funding | 22 | D;V | Give the source of funding and the role of the funders for the present study. | **NA** |

**Supplement Table 6**

The TRIPOD checklist for prediction model development and validation
